# Presentation, management, and outcomes of cauda equina syndrome up to one year after surgery, using clinician and participant reporting: A multi-centre prospective cohort study

**DOI:** 10.1016/j.lanepe.2022.100545

**Published:** 2022-11-17

**Authors:** Julie Woodfield, Ingrid Hoeritzauer, Aimun A.B. Jamjoom, Josephine Jung, Simon Lammy, Savva Pronin, Cathal J. Hannan, Anna Watts, Laura Hughes, Richard D.C. Moon, Stacey Darwish, Holly Roy, Phillip C. Copley, Michael T.C. Poon, Paul Thorpe, Nisaharan Srikandarajah, Gordan Grahovac, Andreas K. Demetriades, Niall Eames, Philip J. Sell, Patrick F.X. Statham, UCES Collaborators, Mohamed Abdelsadg, Motaz MS Abulaila, Usman Ahmed, Qasim Ajmi, Rafid Al-Mahfoudh, Chadi Ali, Meriem Amarouche, Amin Andalib, Mohit Arora, Mukul Arora, Mariam Awan, Afsand Baig Mirza, Antony Bateman, Iwan Bennett, Imran Bhatti, Peter Bodkin, Lalasa Bommireddy, George Bonanos, Anouk Borg, Alexandros Boukas, James Bourne, Rachael Brennan, Jennifer Brown, Katie Brown, Oliver Burton, Christopher Busby, Neil Chiverton, Simon Clark, Phillip C Copley, Simon Cudlip, Yan Cunningham, Ronan Dardis, Stacey Darwish, Benjamin Davies, Andreas K Demetriades, Saurabh Deore, Chris Derham, Muhammad Dherijha, Gareth Dobson, James Duncan, Andrew Durnford, Alexander ZE Durst, Edward W Dyson, Niall Eames, Ellie Edlmann, Andrew Edwards-Bailey, Anne Elserius, Becca Elson, Mohammed Fadelalla, Daniel M Fountain, Adrian Gardner, Arnab Ghosh, James R Gill, Stella A Glasmacher, Robin Gordon, Gordan Grahovac, Rebecca Grenfell, Awais Habeebullah, Nikolaos Haliasos, Tim Hammett, Cathal John Hannan, Ciaran Scott Hill, Ingrid Hoeritzauer, David Holmes, Kismet Hossain-Ibrahim, Laura Hughes, Muhammad Hussain, Shakir Hussain, Ramez Ibrahim, Aimun AB Jamjoom, Bethan John, Shabin Joshi, Josephine Jung, Oliver Kennion, Muhammad Khan, Adriana Klejnotowska, Ashwin Kumaria, Roberta LaCava, Simon Lammy, Alistair Lawrence, Matthew Lea, Andraay HC Leung, Ignatius Liew, Weisang Luo, Oscar MacCormac, James Manfield, Richard Mannion, Joseph Merola, Pranav Mishra, Khalid Abubaker Mohmoud, Richard Moon, Rory Morrison, Odhran Murray, Ali Nader-Sepahi, Colin Nnandi, Anand Pandit, Nitin Patel, Anita Philip, Michael TC Poon, Kuskoor Seethram Manjunath Prasad, Savva Pronin, Shyam Pujara, Balaji Purushothaman, Kapil Rajwani, Fahid Tariq Rasul, Holly Roy, Ahmed-Ramadan Sadek, Moritz Schramm, Gabrielle Scicluna, Philip J Sell, Roozbeh Shafafy, Himanshu Sharma, Asim Sheikh, Vinothan Sivasubramaniam, Agbolahan Sofela, George Spink, Nisaharan Srikandarajah, Patrick FX Statham, Stuart Stokes, Euan Strachan, Chrishan Thakar, Gopiga Thanabalasundaram, Paul Thorpe, Christian Ulbricht, Anna Watts, Alison Whitcher, David White, Kathrin Whitehouse, Martin Wilby, Julie Woodfield, Ardalan Zolnourian, British Neurosurgical Trainee Research Collaborative

**Affiliations:** a Department of Clinical Neurosciences, NHS Lothian, Edinburgh, UK; b Centre for Clinical Brain Sciences, University of Edinburgh, Edinburgh, UK; c Department of Neurosurgery, Aberdeen Royal Infirmary, Aberdeen, UK; d King's College Hospital, London, UK; e Institute of Psychiatry, Psychology & Neuroscience, King's College, London, UK; f Institute of Neurological Sciences, Queen Elizabeth University Hospital, Glasgow, UK; g Southwest Neurosurgical Centre, Derriford Hospital, Plymouth, UK; h University of Plymouth, Plymouth, UK; i The Walton Centre, Liverpool, UK; j Sheffield Teaching Hospitals NHS Foundation Trust, Sheffield, UK; k Department of Neurosurgery, Southmead Hospital, North Bristol NHS Trust, Bristol, UK; l Department of Orthopaedics, Royal Victoria Hospital, Belfast, UK; m Musgrove Park Hospital, Taunton, UK; n University Hospitals of Leicester NHS Trust, Leicester, UK

**Keywords:** Cauda equina syndrome, Back pain, Urinary retention, Cohort study, Spinal surgery

## Abstract

**Background:**

Cauda equina syndrome (CES) results from nerve root compression in the lumbosacral spine, usually due to a prolapsed intervertebral disc. Evidence for management of CES is limited by its infrequent occurrence and lack of standardised clinical definitions and outcome measures.

**Methods:**

This is a prospective multi-centre observational cohort study of adults with CES in the UK. We assessed presentation, investigation, management, and all Core Outcome Set domains up to one year post-operatively using clinician and participant reporting. Univariable and multivariable associations with the Oswestry Disability Index (ODI) and urinary outcomes were investigated.

**Findings:**

In 621 participants with CES, catheterisation for urinary retention was required pre-operatively in 31% (191/615). At discharge, only 13% (78/616) required a catheter. Median time to surgery from symptom onset was 3 days (IQR:1–8) with 32% (175/545) undergoing surgery within 48 h. Earlier surgery was associated with catheterisation (OR:2.2, 95%CI:1.5–3.3) but not with admission ODI or radiological compression. In multivariable analyses catheter requirement at discharge was associated with pre-operative catheterisation (OR:10.6, 95%CI:5.8–20.4) and one-year ODI was associated with presentation ODI (r = 0.3, 95%CI:0.2–0.4), but neither outcome was associated with time to surgery or radiological compression. Additional healthcare services were required by 65% (320/490) during one year follow up.

**Interpretation:**

Post-operative functional improvement occurred even in those presenting with urinary retention. There was no association between outcomes and time to surgery in this observational study. Significant healthcare needs remained post-operatively.

**Funding:**

DCN Endowment Fund funded study administration. Castor EDC provided database use. No other study funding was received.

**Research in context**

**Evidence before this study**

We searched Medline from inception to January 2018 using the term ‘cauda equina syndrome’. We also searched current and previous reports, statements and guidelines from spinal societies and NHS bodies and kept searches up to date during the study. A systematic review of the signs and symptoms of CES identified many literature definitions and concluded that one or more of: bladder, bowel, or sexual dysfunction, reduced saddle sensation, or lower limb neurological deficit should be present for diagnosis. One systematic review of 322 patients used multiple logistic regressions and concluded that sensory and motor deficit, urinary incontinence and rectal dysfunction were more likely to improve with decompression within 48 h. However, timing of symptom onset was defined differently or not defined in many included studies, outcomes were based on few (usually <50) patients per category, and meta-analysis methods were not used. Multiple further systematic reviews of small numbers of patients (up to 200) across studies heterogenous for outcome measures, diagnosis, and measurement of timing, have offered conflicting results concerning whether a shorter time to surgery leads to improved outcomes. Many of these systematic reviews identified that outcomes depend on severity at presentation, with bladder dysfunction at follow up dependent on bladder function at time of surgery. Timing of surgery is regarded in the literature as less important for those in urinary retention as chances of recovery are poor.

**Added value of this study**

This prospective cohort of 621 patients with CES is the largest and most well described cohort published. Our cohort includes a range of severity and symptom types. Of those with a post void residual volume documented on bladder scanning, 59% had a pre-operative residual volume less than 200 mls. Urinary retention requiring catheterisation occurred pre-operatively in 31%. Median time from symptom onset to surgery was 3 days (IQR:1–8), and 32% underwent surgery within 48 h of symptom onset. Earlier surgery was associated with pre-operative catheterisation but not pre-operative disability or degree of radiological compression. At discharge 13% required a catheter, and this was similar at one year. Catheter use at discharge was associated with pre-operative catheterisation but not time to surgery or radiological compression. Disability at one year was associated with disability at admission, but not time to surgery or radiological compression. Healthcare services other than spinal surgery were required by 65% during the one year follow up, and pain scores, quality of life, and disability remained above those expected in the general population at one year.

**Implications of all the available evidence**

Our cohort adds to the literature suggesting that the most common presentation with CES is back pain, sciatica, urinary dysfunction and saddle numbness. Our findings of improvement at discharge and one year across all symptom types and severity show the potential for recovery following surgery for CES. The majority of those catheterised did not require a catheter at follow up, suggesting a benefit of surgical decompression and rehabilitation even for those with urinary retention. This contradicts the theory that those with retention pre-operatively may not recover. Our large cohort with detailed characterisation allowed multivariable regression analysis that suggested that bladder outcomes and disability are associated with severity at presentation but not with time to surgery. These findings do not support previous suggestions of a 24 or 48 h time window in which surgery leads to better outcomes regardless of presentation. However, the observational nature of our study limits the interpretation. Individualised management may confound the results as earlier surgery was associated with pre-operative catheterisation. As 90% underwent surgery within a day of admission, we cannot comment on whether outcomes are worse with delayed surgery, or whether progressive deterioration without treatment occurs. At one year follow up, our cohort with CES had levels of disability, symptoms, and healthcare use above those expected for a working age population, consistent with previous outcome studies, which suggests comprehensive rehabilitation services are required. This large observational study represents the best evidence in the literature so far regarding presentation and outcomes in CES.

## Introduction

Cauda equina syndrome (CES) occurs when the nerve roots within the lumbosacral spinal canal are compressed, usually due to prolapse of an intervertebral disc.^1^ The clinical syndrome can include saddle sensory changes, bladder, bowel, or sexual dysfunction, or bilateral radicular pain.^2^ Back pain, lower limb pain, and lower limb neurological deficits may also be present.^2^ Surgical decompression aims to halt and possibly reverse progressive neurological deficits,^1^ but CES can still lead to significant morbidity, health and social care requirements, and medico-legal consequences.^3^, ^4^, ^5^

There is no agreed clinical definition of CES.^2^ The range in presentation symptoms and severity can lead to a wide differential diagnosis and the involvement of many different healthcare professionals. No individual features reliably predict cauda equina compression on imaging,^6^ and back pain and urinary dysfunction are common, with a population prevalence of 30–40%.^7^^,^^8^ This leads to fewer than 20% of those investigated for CES having radiological cauda equina compression.^9^^,^^10^

Management guidelines advocate timely investigation and management of CES,^11^^,^^12^ but meta-analyses of small historical case series have reached different conclusions regarding whether an optimum time window for intervention after symptom onset exists.^13^^,^^14^ Investigation and management of CES in England has been identified as a patient safety risk,^15^ and there is uncertainty surrounding criteria for and timing of imaging; criteria, timing, and type of surgical intervention; and recovery expectations.^15^^,^^16^

We aimed to describe the clinical features of those treated for CES across the United Kingdom (UK), and their investigation, management, and outcomes. We investigated the association of presentation and management with outcomes.

## Methods

This was a prospective multi-centre observational cohort study of patients with CES in the UK presenting between 1st June 2018 and 31st May 2019.

### Study registration and approval

The protocol was available from April 2018 at www.bntrc.org.uk, and was published in December 2018.^17^ The study was registered prospectively with ISRCTN (16828522). A favourable ethical opinion was given by the South East Scotland Research Ethics Committee 02 (18/SS/0047) in April 2018. All participating centres provided local R&D or management approval with confirmation of capacity and capability. The STROBE checklist was used in preparation of this manuscript following EQUATOR guidelines.

### Participant selection

The inclusion criteria were: age over 18 years; capacity to provide informed consent; and a clinical diagnosis of CES with radiological cauda equina compression. Clinical CES was defined as any of: altered saddle sensation; bladder dysfunction; bowel dysfunction; sexual dysfunction; or bilateral sciatica.^2^^,^^17^ This could be in association with back pain, unilateral sciatica, or unilateral neurological deficit. We excluded those with a unilateral motor or sensory deficit alone (e.g. foot drop) without clinical CES. Only CES caused by degenerative disc disease is included in this report.

We aimed to recruit participants during their emergency admission by contemporaneous screening of admission, referral, and theatre databases. To ensure complete case ascertainment, at six month intervals each centre also checked all those assigned a new ICD-10 code of G83.4 Cauda Equina Syndrome during the study period. Anyone eligible for the study was invited to participate and added to the study database.

We predicted approximately 600–1000 eligible patients in the UK during one year.^17^ The incidence of CES in the UK was unknown at the time of planning the study,^10^ with published estimates starting at 0.3 per 100,000,^10^ but reports of 981 lumbar decompressions for CES in England in 2010 suggesting a higher estimate of 1.9 per 100,000.^17^ For a population of 66 million, these wide estimates translated to between 198 and 1254 presentations with CES per year. We invited all centres in the UK performing emergency surgery for CES to participate, and estimated we would identify 50–80% of all eligible patients. Recruitment was limited to one year due to the resources required for recruitment and follow up.

### Consent

All participants completing questionnaires provided written informed consent. Clinician-reported anonymised data only was included for those without written consent.

### Data collection

Clinician reported data was collected at admission, discharge, and one year post-operatively. Data was entered into a study specific database by the local investigator at the time of identification and at follow up time points. Data sources were direct interaction with the participant during routine care, medical records, and interaction with other clinical staff members caring for the participant (e.g. physiotherapists, operating surgeon). All local investigators were part of the clinical teams caring for the participants. Clinician entered data only included that collected as part of routine care. For participants identified after discharge, clinician reported data for admission and discharge was entered retrospectively into the database at the time the participant was identified as eligible using the same data sources as above.

Magnetic Resonance Imaging (MRI) scans were reviewed by local investigators on their PACS systems and anonymised data was transferred to the central study team. MRI scans were reviewed centrally by a second reviewer blinded to the local assessment to validate the presence of a large disc prolapse with cauda equina compression, the level of cauda equina compression, percentage of canal occlusion, and visibility of CSF. We aimed to second review over 10% of MRI scans (n > 62). With both raters recording CSF visible in 50% of cases, a null hypothesis value of Cohen's kappa of 0.6, and a type I error of 5%, we required 54 MRI scans to be reviewed for 80% power to detect a Cohen's kappa of 0.8.

Participant reported data was collected via electronic, paper, or telephone questionnaires at admission, discharge, six months and one year post-operatively. Non-responders were contacted twice at one year to check if they wished to continue participation. Outcome measures included numerical rating scores for back and leg pain, the Oswestry Disability Index (ODI),^18^ the neurogenic bowel dysfunction (NBD) score^19^ the short form incontinence questionnaire (SFIQ),^20^ the Arizona sexual experiences scale (ASEX),^21^ and parts of the SF-36.^22^ We evaluated all domains from the Core Outcome Set for CES.^23^

### Data analysis

**Fig. 1:**
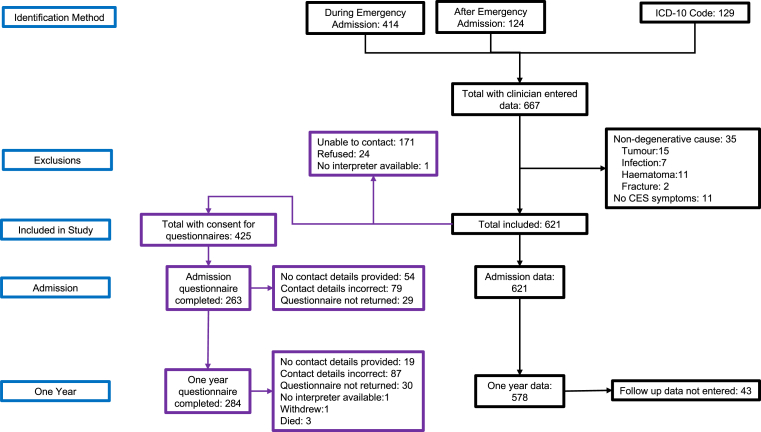
**Study Flow Diagram**. Participant identification, eligibility, inclusion, and reasons for missing follow up data. Black outlines represent clinician entered data. Purple outlines represent participant entered data. Questionnaires and consent were available only in English, but non English speaking participants were included where local interpretation services were available. At one year, participants who had not completed paper or online questionnaires were telephoned to check if they still wished to participate and to check contact details for questionnaires. More detail at each study time point is available in Supplementary Fig. S1 (expanded flow diagram).

Participants with missing data were removed from analyses for which their data were missing, with a complete case analysis performed. Percentages were calculated of those with data available, and the denominator with missing data removed is reported throughout. Some participants did not complete all questions of the follow up questionnaires so the number of participants for each question may be fewer than that shown in Fig. 1. When clinician and participant data was combined (e.g. employment status), participant report was taken first, and clinician reporting used to fill missing data.

We also created variables to represent any pre-operative symptom or sign reported by either patients or clinicians for the main symptom groups. This combined data from multiple responses to identify those with any positive report for the symptom group at any pre-operative time. Catheter use at all time points included indwelling catheters and intermittent self-catheterisation.

We divided participants into categories of CES (early, suspected, incomplete, retention).^17^^,^^24^ To ensure consistency in assigning categories we asked multiple raters to assign categories for a random sample of 100 participants (>10% of the study, sample size as per description for MRI scans) and assessed the inter-rater reliability.

Intervals were calculated in hours where times were available for both start and end points, or days when only dates were available, with one day ranging from less than 1 h up to 48 h. To calculate whether symptom onset to surgery was less than 48 h, intervals in hours were used, then intervals less than two days were counted as less than 48 h and intervals more than two days counted as more than 48 h. For intervals of two days, if a time was given for either symptom onset or operation, and category could be determined, this was used; otherwise the participant was excluded from the category.

ODI index scores and pain scores were calculated,^18^ combined across all time points and plotted as a histogram and a Q–Q plot, with visual confirmation of a normal distribution. ODI and pain scores were compared across groups with t-tests and between time points with Pearson correlation coefficients. Non-parametric numerical data was compared using Mann Whitney U tests. Odds ratios with 95% confidence intervals (CIs) were calculated for categorical data. Inter-rater agreement was assessed using Cohen's kappa for two raters, and Fleiss's kappa for multiple raters.

Multivariable analyses were performed using logistic regression for catheter use and normal bladder function, and linear regression for ODI. Explanatory variables chosen were: age and sex as demographic features measured in all participants, visibility of CSF on MRI as a marker for extent of radiological compression with good inter-rater reliability, catheterisation as a marker of urinary retention and a severe phenotype, time from symptom onset to operation in days as a measure of time to surgery that was available for most participants, and admission ODI as a marker of functional status. Time from symptom onset to operation was chosen as a continuous variable to avoid any preconceptions about 24 or 48 h as important time points. Catheterisation rather than the category of CES with retention was chosen based on the poor inter-rater reliability of assigning patients to the category of CES with retention. We could not include bilateral surgical approaches as an explanatory variable as these were associated with CSF visibility on MRI. We did not include BMI, employment status, or comorbidities as explanatory variables to limit the complexity of the models. The combination of measures of demographic features, imaging, symptom severity and timing were based on possible contributions to outcomes described in previous studies. All analyses were carried out in R version 3.6.3.

### Patient and public involvement

The information leaflet, questionnaires, study aims and data points were developed with feedback from patients with CES.^17^ Patient representatives were not involved in active recruitment of participants as this occurred on an unpredictable emergency basis.

### Role of the funding source

The sponsor and funders played no role in study design; collection, analysis and interpretation of data; writing of the report; or decision to submit for publication.

## Results

We identified 621 participants with clinical cauda equina syndrome and radiological compression of the cauda equina secondary to degenerative disc disease (see Fig. 1 and Fig. S1). Of 389 identified during admission, 352 (90%) consented to questionnaire completion. At one year, 284/425 (67%) of all consented participants returned questionnaires. Of the 45 UK centres providing emergency spinal surgery, 33 (73%) participated. Complete population coverage was achieved in Scotland with all centres performing emergency spinal surgery participating, and ICD-10 coding for the whole country checked. Demographics and clinical findings in the complete Scottish population (n = 149) were similar to those in the overall study (see Table 1). Those returning questionnaires were also similar to the whole study population (see Table 1).

**Table 1:** Demographics and comparison of subgroups.

	Complete Study	Subgroup		
		Scotland (Complete population)	One year questionnaire completed	Pre-operative catheter inserted
	**n = 621**	**n = 149**	**n = 284**	**n = 191**
Age	42 (33–54)	42 (35–53)	43 (35–55)	43 (33–54)
Female	337/621 (54%)	81/149 (54%)	165/284 (58%)	101/191 (53%)
Employed	367/573 (64%)	100/139 (72%)	181/271 (67%)	111/175 (63%)
BMI >30	224/494 (45%)	65/116 (56%)∗	88/229 (38%)∗	83/158 (53%)∗
Comorbidities	307/620 (50%)	72/149 (48%)	143/283 (51%)	95/191 (50%)
**Clinician reported**				
New bladder dysfunction	488/621 (79%)	126/149 (85%)	224/284 (79%)	181/191 (95%)∗∗
New bowel dysfunction	133/621 (21%)	22/149 (15%)∗	63/284 (22%)	40/191 (21%)
New sexual dysfunction	51/232 (22%)	6/85 (7%)∗∗	19/105 (18%)	8/57 (14%)∗
Altered saddle sensation	416/619 (67%)	109/149 (73%)	200/284 (70%)	129/191 (68%)
No visceral symptoms	14/621 (2%)	1/149 (1%)	6/284 (2%)	0/191 (0%)∗
Catheter at admission	182/615 (30%)	45/149 (30%)	82/281 (29%)	182/191 (95%)∗∗
No CSF visible on axial MRI	290/618 (47%)	67/148 (45%)	133/283 (47%)	91/189 (49%)
Interspinous approach	360/613 (59%)	91/148 (61%)	169/277 (61%)	125/189 (66%)∗
Days from symptom onset to surgery	3 (2–9)	4 (2–8)	3 (1–9)	3 (1–6)∗∗
Surgery within 48 h of symptom onset	175/586 (30%)	39/141 (28%)	85/265 (32%)	79/183 (43%)∗∗
Catheter at discharge	78/616 (13%)	17/149 (11%)	27/284 (10%)∗	57/190 (30%)∗∗
Symptom resolution at discharge	273/608 (45%)	78/149 (52%)∗	125/276 (45%)	70/190 (37%)∗
CSF leak	73/619 (12%)	16/149 (11%)	38/282 (13%)	27/191 (14%)
Re-operation	45/612 (7%)	14/149 (9%)	26/280 (9%)	15/190 (8%)
**Participant reported**				
Admission ODI	63 (24)	65 (21)	63 (25)	69 (25)∗
Admission back pain score	7.6 (2.8)	7.8 (2.8)	7.6 (2.9)	7.9 (3.0)
Admission leg pain score	7.8 (3.0)	7.9 (2.8)	7.7 (3.1)∗	8.0 (3.2)
One year ODI	31 (22)	32 (21)	31 (22)	33 (24)
One year back pain score	4.0 (2.7)	4.2 (2.5)	4.0 (2.7)	4.0 (2.6)
One year leg pain score	3.2 (3.1)	2.9 (2.9)	3.2 (3.1)	3.6 (3.1)
Catheter at one year	34/248 (14%)	6/69 (9%)	34/248 (14%)	22/84 (26%)∗∗
Normal bladder function at one year	122/245 (50%)	39/68 (57%)	122/245 (50%)	32/81 (40%)∗

For the complete study, the denominator represents those with data available for that data point. Scotland is a complete population where all patients with CES in Scotland were identified during the study as all centres providing emergency spinal surgery participated and all coding data was screened. Participants from Scotland are compared to participants from the rest of the United Kingdom. Those completing one year questionnaires are compared to a single group of those who did not respond to questionnaires, those not able to be contacted, and those never recruited for questionnaires. Those with a pre-operative catheter inserted are compared to those without catheter insertion.The category ‘no visceral symptoms’ refers to those included due to bilateral sciatica but without bladder, bowel, or sexual dysfunction or altered saddle sensation. Data are median (interquartile range) for age and days from symptom onset to surgery, mean (standard deviation) for ODI and pain scores, and count (percentage) for categorical data. Comparisons between those in the subgroup and those not in the subgroup were made using Mann Whitney U tests for age and days from symptom onset to surgery, t-tests for ODI and pain scores, and chi-square tests for categorical data. (BMI: body mass index, ODI: Oswestry Disability Index, CSF: cerebrospinal fluid, MRI: magnetic resonance imaging, ∗:p < 0.05, ∗∗:p < 0.001 - for difference between those in the subgroup and those not in the subgroup).

### Presentation

**Fig. 2:**
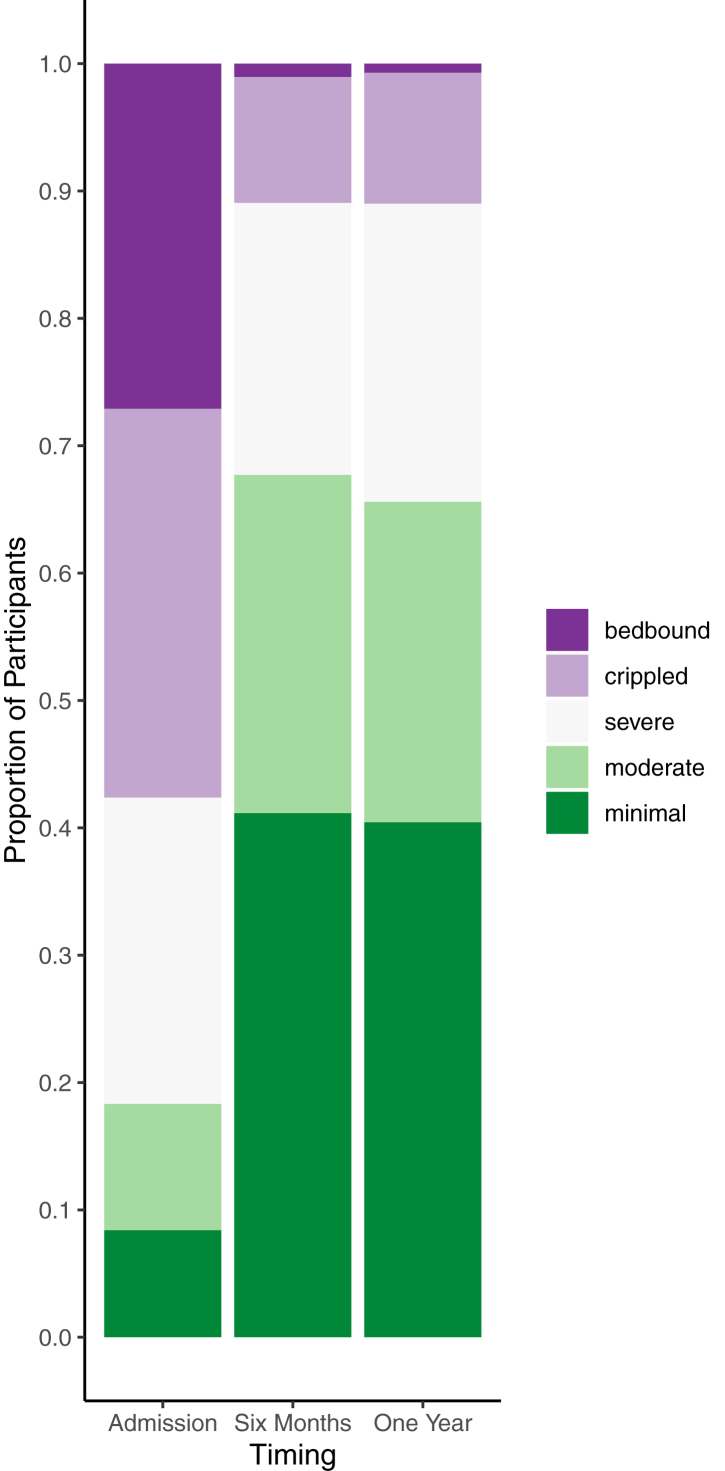
**Oswestry Disability Index**. Oswestry Disability Index (ODI) categories at admission, six months, and one year post operatively. ODI index scores were calculated by dividing the total score by the total possible score of completed items and multiplying by 100. ODI categories are: minimal disability (0–20%), moderate disability (21–40%), severe disability (41–60%), crippled (61–80%), and bed-bound 81–100%). Bars represent all participants completing the ODI at each time point. (Admission: n = 262, mean: 62.5, SD: 24.4; Six months: n = 192, mean:30.4, SD: 21.3; One year: n = 282, mean: 31.1, SD: 22.4). Data for those who returned all three questionnaires were similar (n = 168, Admission: mean: 62.5, SD: 25.7, Six months: mean: 30.2, SD: 21.4, One year: mean: 30.1, SD:21.2).

**Fig. 3:**
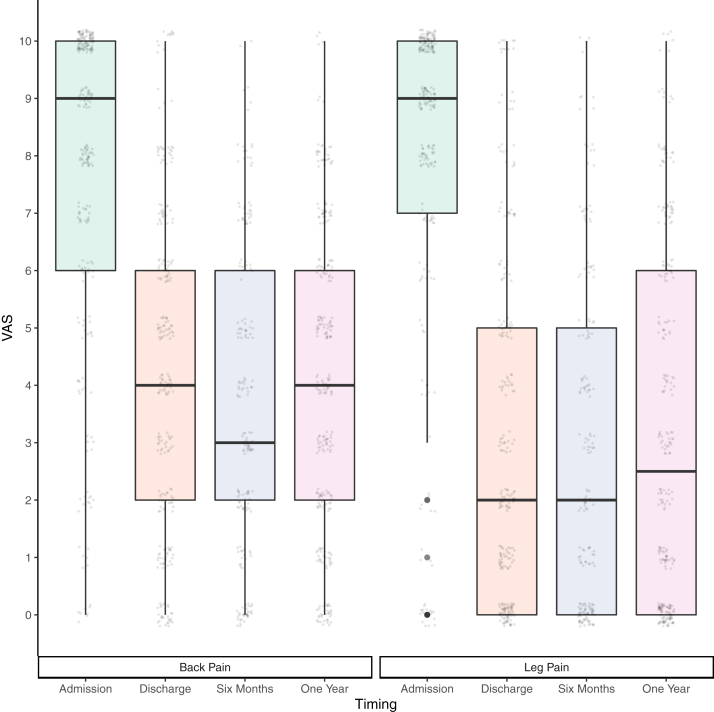
**Pain Scores**. The box plots show pain scores for back pain and worst leg pain at admission, discharge, six months, and one year post-operatively. Circles represent individual pain scores. Participants rated their pain on a scale of 0–10 using an electronic slider or marked on a paper scale. Only whole numbers could be selected. Scores for back pain and pain in the worst leg are shown. All participants who completed pain scores at each time point are shown (back pain: admission = 259, discharge = 253, six months = 193, one year = 278; leg pain: admission = 253, discharge = 253, six months = 187, one year = 272). Pain scores were similar for those completing all four time points (back pain: n = 153, median scores: admission = 9, discharge = 4, six months = 3, one year = 4; leg pain: n = 142, median scores: admission = 9, discharge = 2, six months = 2, one year = 2).

**Fig. 4:**
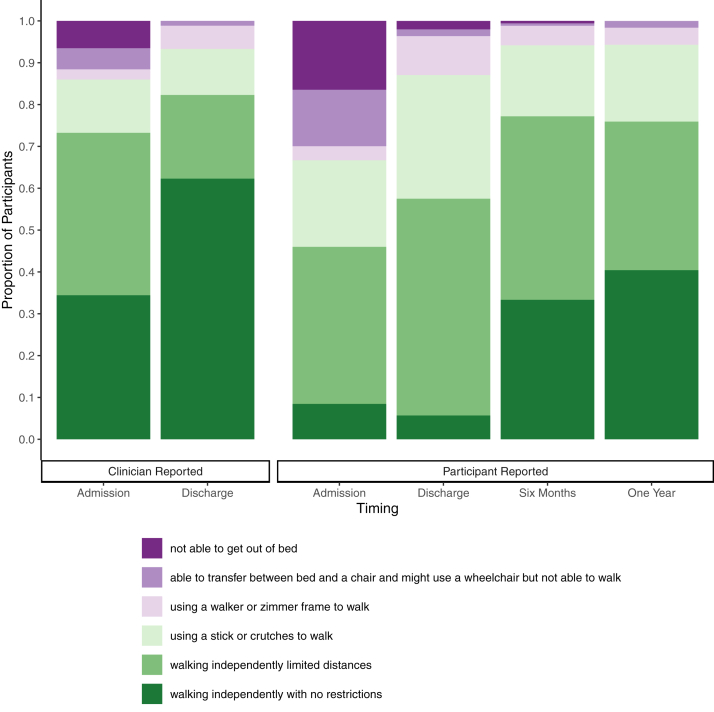
**Mobility**. Mobility is shown at admission, discharge, six months, and one year post-operatively. Both clinicians and participants reported mobility at admission and discharge on the same tick box scale. Bars represent all of those with data available at each time point (clinician reported: admission = 613, discharge = 610; participant reported: admission = 237; discharge = 247; six months = 171; one year = 245). The clinician reported mobility was similar for those with participant reported mobility available and not available (see Supplementary Fig. S2).

The most frequently occurring pre-operative symptoms were back pain (96%, 598/621) and sciatica (93%, 578/621). Participants reported increased morbidity compared to clinicians (see Table 2). Fig. 2, Fig. 3, Fig. 4 show ODI, pain scores, and mobility at admission. Clinician reported pre-operative symptoms and mobility at admission were similar in those completing and not completing questionnaires (see Table 1 and Fig. S2), even though participants reported higher morbidity. Bladder dysfunction (83%, 517/621), and saddle numbness (81%, 502/620) were the most frequent CES symptoms, with bowel dysfunction (39%, 243/621) and sexual dysfunction (38%, 115/300) less frequent. Table S1 shows combinations of symptom types. When bowel and sexual dysfunction were present, bladder dysfunction was frequent (92%, 88%) but of those with bladder dysfunction, it was less common to have bowel (43%) or sexual (42%) dysfunction. Only 2% (14/621) underwent decompression for bilateral sciatica alone without visceral symptoms. Deterioration prior to surgery was reported in 3% (18/619).

**Table 2:** Clinical presentation.

Timing	Admission						Pre-operative	
Source	Clinician reported				Participant reported		Combined participant and clinician reported	
	Symptoms	Missing	Examination	Missing	Symptoms	Missing	Combined	Missing
	n = 621		n = 621		n = 263		n = 621	
**Sciatica**	563/621 (91%)	0	..	..	235/253 (93%)	10	578/621 (93%)	0
Bilateral	258/562 (46%)	1	..	..	160/233 (69%)	12	..	..
**Back pain**	593/621 (95%)	0	..	..	251/259 (97%)	4	598/621 (96%)	0
**Leg weakness**	257/619 (42%)	2	338/621 (54%)	0	..	..	376/621 (61%)	0
Bilateral	82/257 (32%)	2	124/338 (37%)	0	..	..	..	..
Myotomal	108/257 (42%)	3	217/338 (64%)	0	..	..	..	..
KF	11/106 (10%)	4	35/217 (16%)	0	..	..	..	..
KE	9/106 (8%)	4	48/217 (22%)	0	..	..	..	..
AD	88/106 (83%)	4	156/217 (72%)	0	..	..	..	..
AP	53/106 (50%)	4	103/217 (47%)		..	..	..	..
**Leg numbness**	387/620 (62%)	1	443/621 (71%)	0	..	..	481/621 (77%)	0
Bilateral	132/387 (34%)	1	118/443 (27%)	0	..	..	..	..
Dermatomal	231/386 (60%)	2	343/442 (78%)	1	..	..	..	..
L1	2/231 (1%)	2	8/343 (2%)	1	..	..	..	..
L2	4/231 (2%)	2	13/343 (4%)	1	..	..	..	..
L3	18/231 (8%)	2	38/343 (11%)	1	..	..	..	..
L4	77/231 (33%)	2	135/343 (39%)	1	..	..	..	..
L5	156/231 (68%)	2	247/343 (72%)	1	..	..	..	..
S1	143/231 (62%)	2	228/343 (66%)	1	..	..	..	..
**Reflexes**								
KJ reduced/absent	..	..	84/484 (17%)	137	..	..	..	..
Bilateral	..	..	44/84 (52%)	137	..	..	..	..
AJ reduced/absent	..	..	221/465 (48%)	156	..	..	..	..
Bilateral	..	..	120/221 (54%)	156	..	..	..	..
**Mobility**								
Independent, no restrictions	..	..	211/613 (34%)	8	20/237 (8%)	26	170/618 (28%)	3
Independent, limited distance	..	..	238/613 (39%)	8	89/237 (38%)	26	217/618 (35%)	3
Using stick/crutches	..	..	78/613 (13%)	8	49/237 (21%)	26	100/618 (16%)	3
Using walker/zimmer	..	..	15/613 (2%)	8	8/237 (3%)	26	19/618 (3%)	3
Transfers only	..	..	31/613 (5%)	8	32/237 (14%)	26	50/618 (8%)	3
Bed bound	..	..	40/613 (7%)	8	39/237 (16%)	26	62/618 (10%)	3
**SLR restricted**	–	..	231/257 (90%)	364	..	..	..	..
Bilateral	..	..	115/231 (50%)	364	..	..	..	..
**Altered saddle sensation**	416/619 (67%)	2	417/575 (73%)	46	161/229 (70%)	34	502/620 (81%)	1
Bilateral	259/412 (63%)	6	222/417 (53%)	46	..	..	..	..
**Bladder symptoms**	499/621 (80%)		..	..	176/231 (76%)	32	517/621 (83%)	0
New symptoms	488/621 (79%)	0	..	..	..	..	..	..
Retention	205/499 (41%)	0	..	..	22/176 (13%)	32	..	..
Incontinence	201/499 (40%)	0	..	..	40/176 (23%)	32	..	..
Altered sensation	109/499 (22%)	0	..	..	..	..	..	..
Poor stream	187/499 (37%)	0	..	..	..	..	..	..
Urgency/Frequency	47/499 (9%)	0	..	..	..	..	..	..
**Catheterisation**	..	..	182/615 (30%)	6	22/231 (10%)	32	191/615 (31%)	6
New catheter	..	..	181/615 (29%)	6	..	..	..	..
Unable to feel catheter	..	..	31/104 (30%)	84	13/20 (65%)	34	..	..
**Bowel symptoms**	143/621 (23%)	0	..	..	139/230 (60%)	33	243/621 (39%)	0
New symptoms	133/621 (21%)	0	..	..	..	..	..	..
Incontinence	57/143 (40%)	0	..	..	25/139 (18%)	33	..	..
Altered bowel habit	69/143 (48%)	0	..	..	..	..	..	..
Altered sensation	22/143 (15%)	0	..	..	..	..	..	..
**Sexual symptoms**	57/232 (25%)	389	..	..	68/223 (30%)	40	115/300 (38%)	326
New symptoms	51/232 (22%)	389	..	..	..	..	..	..
Altered sensation	23/57 (40%)	389	..	..	..	..	..	..
Difficulty achieving orgasm/erection	36/57 (63%)	389	..	..	..	..	..	..

Admission symptoms were reported by both clinicians and participants. Admission signs were recorded from examination findings by clinicians. Both were asked to record symptoms and signs at the time of admission. Pre-operative findings are positive where either the participant or the clinician has reported that finding at any time point prior to the operation. Bilateral occurrence is opposed to unilateral. Weakness was either myotomal or non-myotomal pattern. Sensory loss was either dermatomal, or non-dermatomal. For bladder symptoms, retention included painful or painless retention, a feeling of incomplete voiding, inability or difficulty voiding, or a loss of awareness of the need to void. Incontinence included all types of incontinence. Altered sensation included an altered sensation of voiding. Poor stream included difficulty initiating urination, dribbling, needing to push, and poor stream. For bowel symptoms, altered bowel habit included constipation or diarrhoea, or increased/decreased frequency of passing stool compared to usual. Altered sensation included the sensation of passing stool and of knowing whether to pass stool. For sexual symptoms, difficulty achieving orgasm or erection includes any physical difficulty with sexual intercourse. Altered sensation includes any change in sensation during sexual intercourse such as altered vaginal sensation, and altered sexual impulses. Participant reported back pain and sciatica is taken from the visual analogue scale (VAS) for back and leg pain, where zero is equivalent to no back or leg pain, and any score higher than zero denotes back or leg pain. Percentages are a proportion of available data. Missing data is excluded on a case wise basis. (KF: knee flexion, KE: knee extension, AD: ankle dorsiflexion, AP: ankle plantarflexion, KJ: knee jerk, AJ: ankle jerk, SLR: straight leg raise).

Bladder scanning was undertaken in 59% (368/621), of whom 59% (216/368) had a post-void residual volume of <200 mls (see Table 3). A catheter was inserted pre-operatively in 31% (191/615). In 181 the catheter was inserted on admission, in one participant the catheter was in place prior to admission, and nine had a catheter inserted after admission but pre-operatively. Of those tested, 30% (31/104) had no sensation to a catheter tug. Those with a catheter inserted were demographically similar to those without (see Table 1).

**Table 3:** Residual volumes.

Residual volume recorded	Modality	
	Bladder scan	Post catheterisation
	n = 368	n = 156
**Volume (mls)**		
<100	172 (47%)	9 (6%)
100-199	44 (12%)	10 (6%)
200-299	47 (13%)	26 (17%)
300-399	18 (5%)	11 (7%)
400-499	19 (5%)	18 (12%)
≥500 mls	68 (18%)	82 (53%)

Post void residual volumes were recorded in millilitres (mls) using ultrasound bladder scanning after the participant had been instructed to void, or post catheterisation by measuring the residual volume drained following insertion of the catheter. Percentages are of the number with a residual volume recorded.

Light touch saddle sensory examination was abnormal unilaterally in 32% (174/541), abnormal bilaterally in 37% (198/541), and normal in 31% (169/541). Pin prick examination was abnormal unilaterally in 35% (141/399), abnormal bilaterally in 32% (128/399), and normal in 33% (130/399). Digital rectal examination was abnormal in 43% (226/524). Abnormal findings were loss of anal tone (164/226, 73%) and loss of internal rectal sensation (78/226, 35%).

We attempted to divide participants into the categories of early CES, suspected CES, incomplete CES and CES with retention.^17^^,^^24^ However, the inter-rater reliability for nine clinicians dividing participants into these categories based on clinical features at presentation was low (κ = 0.31, 95%CI:0.58–0.87). We therefore did not report or use these categories further for analysis, but instead describe individual clinical features.

### Imaging

All participants underwent MRI lumbosacral spine except one who underwent a CT spine without contrast after not tolerating an MRI. Imaging was usually performed prior to admission to the spinal unit (at another hospital site: 52% (319/619), at the same hospital site: 26% (160/619), after admission to the spinal unit: 23% (140/619)). All included participants had significant cauda equina compression with at least 50% canal stenosis due to degenerative disc disease on review by local investigators. This was confirmed in all of the 101 (16%) scans that underwent a second blinded review. More than 75% canal occlusion was present in 70% (430/618), and 47% (290/618) had no CSF visible on axial T2 imaging at the most affected level. Inter-rater agreement between central review and local clinician review was high (κ = 0.78, 95%CI:0.66–0.90) for visibility of CSF and for greater than 75% canal occlusion (k = 073, 95%CI:0.58–0.87) in the 101 (16%) of scans that underwent a blinded second review. The level of compression was: L4/5 (50%, 306/617); L5/S1 (36%, 223/617); L3/4 (11%, 68/617); L2/3 (2%, 15/617); L1/2 (1%, 5/617). Lack of visible CSF on MRI was not associated with mean admission ODI (CSF not visible:61, CSF visible:63, t_233_ = −0.8, 95%CI:-8.4–3.8), or with pre-operative catheterisation (CSF not visible:91/288, 32%, vs CSF visible:98/324, 30%, OR:1.1, 95%CI:0.7–1.5).

### Surgical management

All participants underwent surgical decompression. This was performed by neurosurgeons in 73% (455/621) and orthopaedic surgeons in 27% (166/621). The primary surgeon was a consultant in 28% (124/450) of neurosurgical procedures and 86% (143/166) of orthopaedic procedures. Surgical procedures were single level in 96% (595/618). Interspinous approaches were more frequent when CSF was not visible on MRI (interspinous with CSF not visible:193/287, 67%, vs CSF visible: 51%, OR:1.9, 95%CI:1.4–2.7). Bilateral muscle strip, laminectomy, and discectomy were also more frequent when CSF was not visible on MRI (see Table S2).

### Timing of surgical intervention

**Fig. 5:**
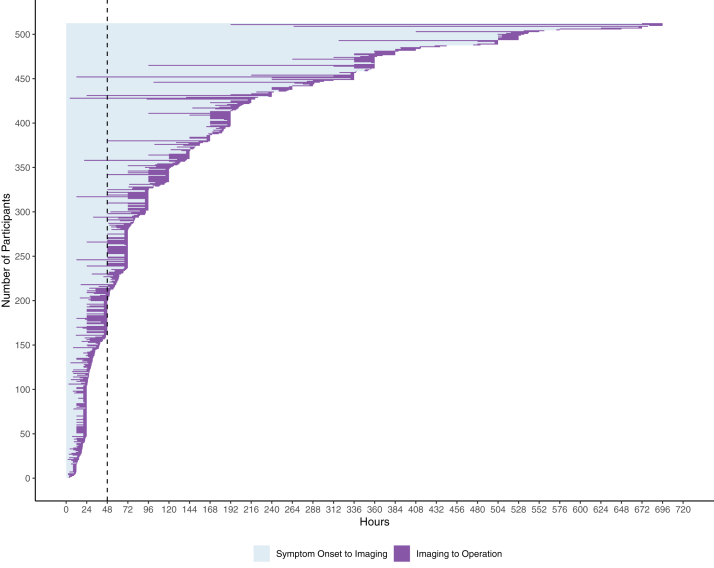
**Time From Symptom Onset To Imaging and Operation**. Time from symptom onset in hours is shown on the x axis. Each bar represents a single participant. Participants are ordered from shortest to longest time to operation. Time to imaging is shown in light blue and time from imaging to operation is shown in purple. The vertical dotted line marks 48 h from symptom onset. Only those undergoing surgery as an emergency or on the next day list are shown. Those undergoing surgery on a planned theatre date are not shown (n = 41). Those undergoing surgery with imaging that was performed prior to the onset of CES symptoms are not shown (n = 24). Times to surgery from symptom onset longer than 30 days (720 h) are not shown (n = 38). Where intervals in hours are available, these are plotted (n = 226). For participants where intervals are only available in days, these have been converted to hours by multiplying by 24, except zero days, which is represented as 12 h to allow visualisation of time to surgery (n = 203). Where intervals were converted from days to hours and hours to imaging and surgery were the same, the time to imaging is set 4 h prior to surgery to allow data visualisation (n = 101).

Urgency of surgery is shown in Table 4. Forty-one planned operations taking place from 2 to 69 days following referral (median: 2 days, IQR: 2–4) were excluded from analyses of time to operation. Surgery was performed within one day of admission in 99% (570/578). and within one day from referral in 90% (518/577). Median time to surgery was 3 days (IQR:1–8) from clinician reported symptom onset and 5 days (IQR:2–34) from participant reported symptom onset (see Fig. 5 & Fig. S3). Surgery was performed within 48 h of clinician reported symptom onset in 32% (175/545). There were 31 patients with dates of symptom onset and surgery who underwent surgery on the second day after symptom onset, but it could not be determined if this was before or after 48 h.

**Table 4:** Timing and urgency of surgery.

	Emergency	Next day list	Planned list
Total	405/619 (65%)	173/619 (28%)	41/619 (7%)
In hours	109/404 (27%)	141/173 (82%)	36/41 (88%)
Out of hours	295/404 (73%)	32/173 (18%)	5/41 (12%)
Unknown	1	0	0
Normal Hours	250/380 (66%)	173/173 (100%)	41/41 (100%)
Early hours	130/380 (34%)	0	0
Unknown	25	0	0

Urgency of surgery was categorised as emergency, on the next day's list, or on a planned list. A planned list was any list that was after the next day's list. Out of hours operations were defined as those performed at any time on a Saturday or Sunday, those starting after 18:00 or before 08:00 or where no start time was available but the clinician had stated it was an out of hours operation. In hours operations were those performed Monday to Friday starting between 08:00 and 18:00 or when no start time was available but the clinician stated it was performed in hours. Early hours operations were defined as operations starting after 23:00 and prior to 07:00 on any day of the week. Normal hours were any operations not performed in the ‘early hours’ times. Urgency of operation was unknown in two cases. Planned operations could also be out of hours if they were performed during the day at weekends.

At the time of referral to the spinal unit, only 39% (223/575) had symptom onset within one day, and 77% (443/575) within one week. First contact with a healthcare professional was reported by participants within one day of symptom onset in 58% (116/201) and within a week in 78% (156/201). Participants most often first contacted their General Practitioner (GP) (56%, 140/250), followed by telephone triage systems (20%, 49/250), then Emergency Departments (ED) (18%, 46/250). However, referrals to spinal units came most frequently from EDs (55%, 340/615), and only 3% (8/263) participants reported seeing only their first point of contact prior to surgery.

Surgery within 48 h of symptom onset was associated with catheterisation (≤48hrs:79/174, 45% vs > 48hrs:100/367, 27%, OR:2.2, 95%CI:1.5–3.3) and emergency rather than next day surgery (≤48hrs:154/175, 88% vs > 48hrs:233/370, 63%, OR:4.3, 95%CI:2.6–7.5), but not with admission ODI (mean:≤48hrs:64 vs > 48hrs:62, t_136_ = −0.7 95%CI:-9.5–4.3), or lack of visible CSF (≤48hrs:89/175, 51%, vs > 48hrs:164/367, 45%, OR:1.3, 95%CI:0.9–1.9).

### Outcomes

Clinicians reported complete symptom resolution at discharge in 45% (273/608), with ongoing back pain in 18% (110/608) and ongoing sciatica in 8% (46/608). Participant reported pain scores were zero at discharge in only 11% (27/253) for back pain and 27% (69/253) for leg pain. Pain scores were lower at discharge compared to admission, and remained similar at one year (see Fig. 3). Leg weakness at one year was reported by 66% (188/284), and abnormal sensation to a non-painful stimulus by 51% (125/244). Both participants and clinicians reported improved mobility post-operatively (see Fig. 4), but participants consistently reported worse mobility than clinicians (Fig. 4 and Fig. S2).

### Bladder function

Bladder symptoms at discharge were reported by 49% (117/241) participants, but only in 17% (106/608) of cases by clinicians. At one year, 50% (122/245) reported abnormal bladder function, with 34% (83/242) not always able to tell when their bladder was full. SFIQ results are in Figs. S4 and S5. Of those who were catheterised pre-operatively, 30% (57/190) required a catheter at discharge, and 26% (22/84) at one year (see Table 5).

**Table 5:** Catheter use.

	Clinician	Participant	Clinician & participant
Admission	182/615 (30%)	22/231 (10%)	..
Pre-operative	184/615 (30%)	..	191/615 (31%)
Discharge	78/616 (13%)	25/241 (10%)	78/616 (13%)
Six Months	..	20/170 (12%)	..
One Year	..	34/248 (14%)	..

Catheter use as reported by clinicians and participants. Catheter use includes indwelling catheter on free drainage, indwelling catheter with flip-flow valve, and intermittent self-catheterisation. Pre-operative catheterisation includes anyone reported as catheterised prior to the operation by either the clinician or the participant. At admission, seven participants reported catheterisation that was not reported by clinicians. At discharge, none of the participants reported catheterisation who were not reported to be catheterised by the clinician.

Catheter use at discharge was not associated with lack of visible CSF on MRI (catheter:33/77, 43%, vs no catheter:255/537, 47% OR:0.8, 95%CI:0.5–1.4), or admission ODI (mean:catheter:70, no catheter:62, t_31_ = −1.7, 95%CI:-19.1-1.5), but was associated with pre-operative catheterisation (catheter:57/75, 76%, vs no catheter:133/535, 25%, OR:9.6, 95%CI:5.3–17.8), lack of sensation to the catheter pre-operatively (catheter:15/35, 43%, vs no catheter: 16/69, 23%, OR:2.5, 95%CI:0.9–6.5) and operation within 48 h of symptom onset (catheter:31/64, 48%, vs no catheter:142/476, 30%, OR:2.2, 95%CI:1.3–3.9). In multivariable logistic regression modelling for catheter use at discharge, only pre-operative catheterisation was associated with discharge catheterisation (adjusted OR:10, 95%CI: 5.8–20.4, see Table 5). Normal bladder function at one year was associated with male sex, not requiring a catheter pre-operatively and a lower admission ODI (Female: adjusted OR: 0.3, 95%CI:0.2–0.7; Catheter inserted: adjusted OR: 0.5, 95%CI: 0.3–1, admission ODI adjusted OR:0.98, 95%CI:0.97–1.0, see Table 6).

**Table 6:** Multivariable adjusted odds ratios for outcome measures.

Outcome	Timing of Outcome		
	Discharge	One Year	
	Catheter Use	Normal Bladder Function	ODI Score
**Participants in model**	n = 565	n = 169	n = 200
**Explanatory variables**			
Age	1.00 (0.98–1.02)	1.0 (0.98–1.03)	0.15 (−0.05 to 0.35)
Female	0.98 (0.56–1.72)	*0.33 (0.17-0.65)∗*	*10.92 (5.29-16.54)∗∗*
CSF not visible on axial MRI	0.75 (0.43–1.31)	1.00 (0.52–1.94)	−1.25 (−6.68 to 4.18)
Catheter inserted pre-operatively	*10.56 (5.81-20.41)∗∗*	0.50 (0.24–1.02)∗	3.15 (−2.76 to 9.06)
Days from symptom onset to operation	0.99 (0.96–1.01)	1.01 (0.98–1.04)	0.09 (−0.12 to 0.30)
Admission ODI Score	..	*0.98 (0.97-1.00)∗*	*0.28 (0.17-0.40)∗∗*

Multivariable logistic regression for the outcomes of catheter use at discharge and normal bladder function at one year was performed. Adjusted odds ratios with 95% confidence intervals for predictors are shown. Admission ODI and catheter tug findings were not included in the model for discharge catheter use due to the small numbers of participants with complete data. Similar numbers of participants had complete data for ODI at admission and normal bladder function at one year, so ODI at admission was included in the model for normal bladder function. Linear regression was used for the ODI index score at one year. Regression coefficients with 95% confidence intervals are shown for explanatory variables. The measure of time to operation chosen was symptom onset to operation in days as this was the most complete. Those undergoing a planned operation were excluded from the model. (ODI: Oswestry Disability Index, CSF: cerebrospinal fluid, ∗p < 0.05, ∗∗p = <0.001).

### Bowel function

Abnormal bowel function was reported by 49% (118/239) participants at discharge, and 43% (104/241) at one year. Clinicians reported only 9% (12/140) of those with bowel symptoms at admission had ongoing bowel symptoms at discharge. NBD categories at one year are in Fig. S4.

### Sexual function

More participants rated their sexual function as normal at one year (105/237, 44%) compared to on admission (56/223, 25%). 51% (127/250) had sexual dysfunction at one year on the ASEX (see Fig. S4).

### Saddle sensation

Saddle sensation was normal at discharge in 45% (108/240) and at one year in (54%, 130/241) participants.

### Functional outcomes

At one year, 56% (134/239) were employed. Of those employed at admission, 79% (118/150) remained employed at one year. SF-36 scores at one year are shown in Fig. S5. Mean scores (with 100 being most favourable) were 53(SD:26) for general health, and 61(SD:23) for emotional well-being.

ODI index scores improved (decreased) from a mean of 63(SD:24) at admission to 31(SD:22) at one year (see Fig. 2 and Figs. S4 and S5).

ODI at one year was associated with ODI at admission (*r* = 0.4, 95%CI:0.3–0.5), but not with lack of CSF visible on MRI (mean:CSF not visible:29, CSF visible:33, t_278_ = −1.3, 95%CI:-8.7–1.74), pre-operative catheterisation (mean:catheter:33, no catheter:30, t_157_ = −0.8, 95%CI:-8.3-3.5), or operation within 48 h of symptom onset (mean ≤ 48hrs: 31, >48hrs:30, t_167_ = −0.5, 95%CI:-6.3-5.2). In multivariable analysis, a higher (worse) ODI at one year was associated with a higher ODI at admission (t = 0.3, 95%CI:0.2–0.4) and female sex (t = 10.9, 95%CI:5–17), but not time to operation (t = 0.09, 95%CI:-0.1–0.3, see Table 6).

### Health service use

The median length of hospital inpatient stay was 3 days (IQR:2–5), and 94% (575/609) were discharged to their usual place of residence. Two thirds of participants required healthcare services other than spinal surgery in the post-operative year (see Supplementary Table S4).

### Complications

Complications occurred in 26% (160/619) participants. These included durotomy or CSF leak (12%, 73/619), neurological worsening post-operatively (12%, 76/619), medical complications (4%, 26/619), and wound problems (2%, 12/619). Re-operation within one year occurred in 7% (45/612). This was due to recurrent/residual disc (35), infection (4), haematoma (3), CSF leak (1), or for instrumentation (2). Re-operation occurred within 2 weeks in 49% (22/45).

## Discussion

This is the largest prospective study of patients with CES and one of few with patient reported outcomes. The most common presentation was back (96%) and leg pain (93%) with urinary dysfunction (83%) and saddle numbness (81%). Lack of CSF visibility on axial MRI (a marker of radiological compression) was not associated with severity of presentation (measured using the ODI) or pre-operative urinary retention requiring catheterisation. Most (59%) participants with CES had a post void residual volume of less than 200 mls pre-operatively. Surgery was undertaken within 48 h of presentation in 32%. Those requiring catheterisation (31%) underwent surgery sooner. The requirement for a catheter at discharge (13%) was associated with pre-operative catheterisation, but not with time to surgery, or lack of CSF visibility. Worse ODI at one year was associated with worse pre-operative ODI, but not with time to surgery or lack of CSF visibility. Two-thirds required healthcare services other than spinal surgery in the year following surgery.

The strength of this study is the large cohort with comprehensive data on presentation, management and outcomes that allows multivariable associations with outcomes to be assessed. We covered the complete 5.4 million population of Scotland, and findings in Scotland were similar to the rest of the UK (Table 1). The range of presentations described in Table 2 is similar to that seen in UK practice. Therefore our study population is likely representative of patients presenting with CES in the UK. Participant reporting is a strength of our study. Participants consistently reported higher morbidity than clinicians, and also reported a longer time from symptom onset to operation. Although participant reporting can lead to self-report bias, it is likely that clinicians also underreport, and it is the experience of participants that determines quality of life rather than objective findings.

The study is limited by incomplete participant reported outcomes (n = 284) at one year, and missing data throughout the study. Although the response rate is high (284/371, 77%) for those consented during their emergency admission, the lower overall rate reflects the challenges of in-hospital study recruitment for an emergency condition with a median length of stay of 3 days. Although most (81%, 538/667) were identified from their emergency admission and followed prospectively, the remainder were identified later with retrospective data entry up to the time of identification. Clinician data was mostly missing due to it not being routinely acquired, for example, reflexes were not examined or sexual function was not discussed. This is likely clinician dependent missing data that is missing at random, but we cannot exclude that symptoms and signs were more likely to be documented when it was felt there was a higher chance of abnormality. Those providing questionnaire responses were similar to those without responses (Table 1). There was no evidence that only those with more severe sequalae responded, as the rate of catheterisation at discharge was slightly lower in those who did respond (10% vs 13%, see Table 1). As predictors such as catheterisation and ODI were included in the multivariable models, we did not analyse missing data further. Other limitations of this study are that the findings are only relevant to those with CES due to degenerative disc disease, and may only be relevant to those treated in the UK health system. Although we assessed all domains of the recently developed COS for CES,^23^ there is not as yet a standard method of measuring these domains. Methods of reporting and different definitions could lead to different conclusions regarding effects of interventions on outcomes.

There are few other studies of CES using participant reported outcome measures for comparison. Our ODI scores at one year (mean:31, SD:22) were higher (worse) than in the general population (mean:10, range:2–12),^25^ and higher than one year post-operative scores following discectomy for sciatica (mean pre-op:47.5, mean decrease:-30.6).^26^ SF-36 scores at one year (general:53, SD:26; emotional:61, SD:23) were also worse (lower) than population normative scores (general:74, SD:22; emotional:88, SD:29),^27^ and worse than one year post discectomy scores (general: 74.2, SE:1.8; emotional:87.2, SE:2.6).^28^ Our mean pre-operative back pain score of 7.6/10 decreasing to 4.0/10 at one year compares to a mean pre-operative back pain score of 34/100 decreasing to 17/100 at one year following discectomy for sciatica.^28^ The absolute magnitude of the one year post-operative change in ODI, SF-36, and pain scores from pre-operative levels for CES is therefore similar to that seen following discectomy for sciatica, albeit from worse pre-operative values.^26^^,^^28^ Ongoing post-operative pain, poorer than expected quality of life, and need for healthcare services highlights the need for comprehensive rehabilitation and support services following CES.

In multivariable analyses, we found no association of outcome with time from symptom onset to surgery. Assessment of the influence of time to surgery is limited by the observational nature of this study where timing was determined by patient presentation, healthcare system, and treating clinician. Surgery was undertaken more quickly in those requiring catheterisation pre-operatively, and pre-operative catheterisation was associated with post-operative catheter requirement. Outcomes may also be influenced by type of management, and surgical technique was associated with visibility of CSF on MRI. This may further confound any association between timing of intervention and outcome. Previous meta-analyses of time to surgery with different methods have led to different conclusions regarding the effect of timing on outcomes and whether surgery within 24 or 48 h confers an advantage, and to whom.^1^^,^^13^^,^^14^ Lack of association of outcomes with time to surgery within a system where surgery is undertaken quickly after diagnosis is consistent with previous small observational cohort studies,^4^^,^^5^^,^^29^ but none described more than 140 participants so adjustment for presentation and management was not possible. Defining time of symptom onset can be challenging in a condition with multiple changing symptoms and no clear definition, and differing start points have been used in previous studies. This prospective, large, observational cohort study is the highest level of evidence possible for CES, where randomisation to treatment delay is unlikely to occur. Our findings do not support better outcomes with surgery undertaken within 48 h of symptom onset, within this contemporary cohort where 90% underwent surgery within one calendar day of referral to spinal services. As outcomes were associated with severity and type of symptoms at presentation, there is however, an argument for more urgent surgery in those who are rapidly deteriorating.

At discharge, 70% of those catheterised pre-operatively, and 57% of those without sensation to the catheter were no longer using a catheter. This contradicts previous opinion that those in retention or with an insensate bladder may not benefit from early surgical decompression due to lack of potential for recovery.^1^ However, comparison with historical studies is complex, as different criteria for catheterisation may have been applied and different definitions of CES with retention have been described.^1^ Our catheterised sample includes those with sensation to the catheter, so may not be as severe as those previously described with completely insensate bladders.

In this cohort with CES, 59% of the 368 who underwent pre-operative post void bladder scans had a residual volume of less than 200 mls, and 47% had a residual volume less than 100 mls. As we did not include those investigated without CES, we cannot comment on the ability of bladder scanning to discriminate between those with or without radiological cauda equina compression. However, it is difficult to support the use of bladder scanning to determine access to MRI with this data.

In conclusion, this is the first study that has successfully followed patients with CES across a country from their emergency presentation, and collected detailed presentation, management and outcome data. This study is the highest level of evidence for CES currently available. There was no association between outcomes studied and time from symptom onset to surgery in this cohort where most underwent decompression within a day of diagnosis and referral at a median of 3 days following symptom onset. At discharge 70% of those catheterised pre-operatively no longer needed a catheter, suggesting there is potential for recovery in catheterised patients. Participant reported outcome data suggested significant ongoing disability and symptoms following CES highlighting the need for rehabilitation services.

## Contributors

Study conceptualisation and methodology: JW, IH, SP, AKD, PFXS, PJS, NE, NS; data curation, formal analysis, visualisation: JW, IH, AABJ, SP, MP, HR; resources and funding acquisition: JW, IH, AABJ, SP, PFXS; investigation and data collection: JW, AABJ, JJ, SL, HR, PCC, CJH, AW, LH, RDCM, SD, PT, GG, NE, SP; project administration: JW, IH, AABJ, SP; writing of draft manuscript: JW, MTCP, IH, AABJ, SP, NS; review and editing of draft: JW, IH, AABJ, MTCP, JJ, SL, HR, PCC, NS, CJH, AW, LH, RDCM, SP, SD, PT, AKD, GG, NE, PJS, PFXS. JW, SP, AABJ, and MTCP have directly accessed and verified the underlying data reported in the manuscript. JW acts as guarantor for the data and analysis.

## Data sharing statement

Anonymised individual participant data and a data dictionary will be available following approval of a proposal submitted to the steering committee via the chief investigator (julie.woodfield@ed.ac.uk) with a data access agreement for ethically approved studies.

## Declaration of interests

JW, AABJ, JJ, SL, SP, CJH, AW, LH, RDCM, SD, HR, PCC, MTCP, NS, GG, NE, and PJS declare no conflicts of interest during the study or within 3 years of the work being submitted. IH declares support for attending meetings and payment or honoraria for speaking about functional neurological disorders (including persistent postural perceptual dizziness) at conferences and meetings. IH has received payment for expert testimony on idiopathic urinary retention. PFXS has received payment for expert testimony, acting for a number of both claimants and defenders in cases of Cauda Equina Syndrome, roughly in the proportion 2/3 defender, 1/3 claimant over about 20 years. PT has received payment for expert testimony for Cauda Equina Syndrome cases for DAC Beachcroft, Aspire Law, Bevan Brittan LLP, Stephensons LLP, Moore Barlow Ltd, Scott Rees & Co, and Premex/Premex +. AKD declares payment or honoraria for speaking for Integra, Stryker, and Safe Orthopaedics. AKD declares leadership board roles (unpaid) for Global Neuro Foundation and European Association of Neurosurgical Societies.
