# Supplementary material for: Presentation, management, and outcomes of cauda equina syndrome up to one year after surgery, using clinician and participant reporting: A multi-centre prospective cohort study
Source: Lancet Reg Health Eur. 2022 Nov 17;24:100545. doi: 10.1016/j.lanepe.2022.100545 (PMC9678980; doi:10.1016/j.lanepe.2022.100545)
Supplement: Supplementary File S1 [file mmc1.pdf]

Supplementary Information

Contents:

1. **Supplementary Figures**
2. **Supplementary Tables**
3. **List of Collaborators**
4. **Participant Information Sheet and Consent Form**
5. **Study Protocol**

1. Supplementary Figures

Supplementary Figure 1

Complete Study Flow Diagram

Expanded study flow diagram accounting for all participants at each time point. Participant identification, eligibility, exclusions, and reasons for missing follow up data are shown. Black outlines represent clinician entered data.Purple outlines represent participant entered data. Questionnaires were marked as not returned when contact details were correct and participants could be contacted by telephone, email, or post, but did not return questionnaires. Questionnaires and consent were available only in English, and non English speaking participants were included at time points only where local interpretation services were available. Clinician entered discharge and one year follow up data was not entered when local centres were unable to provide this information.

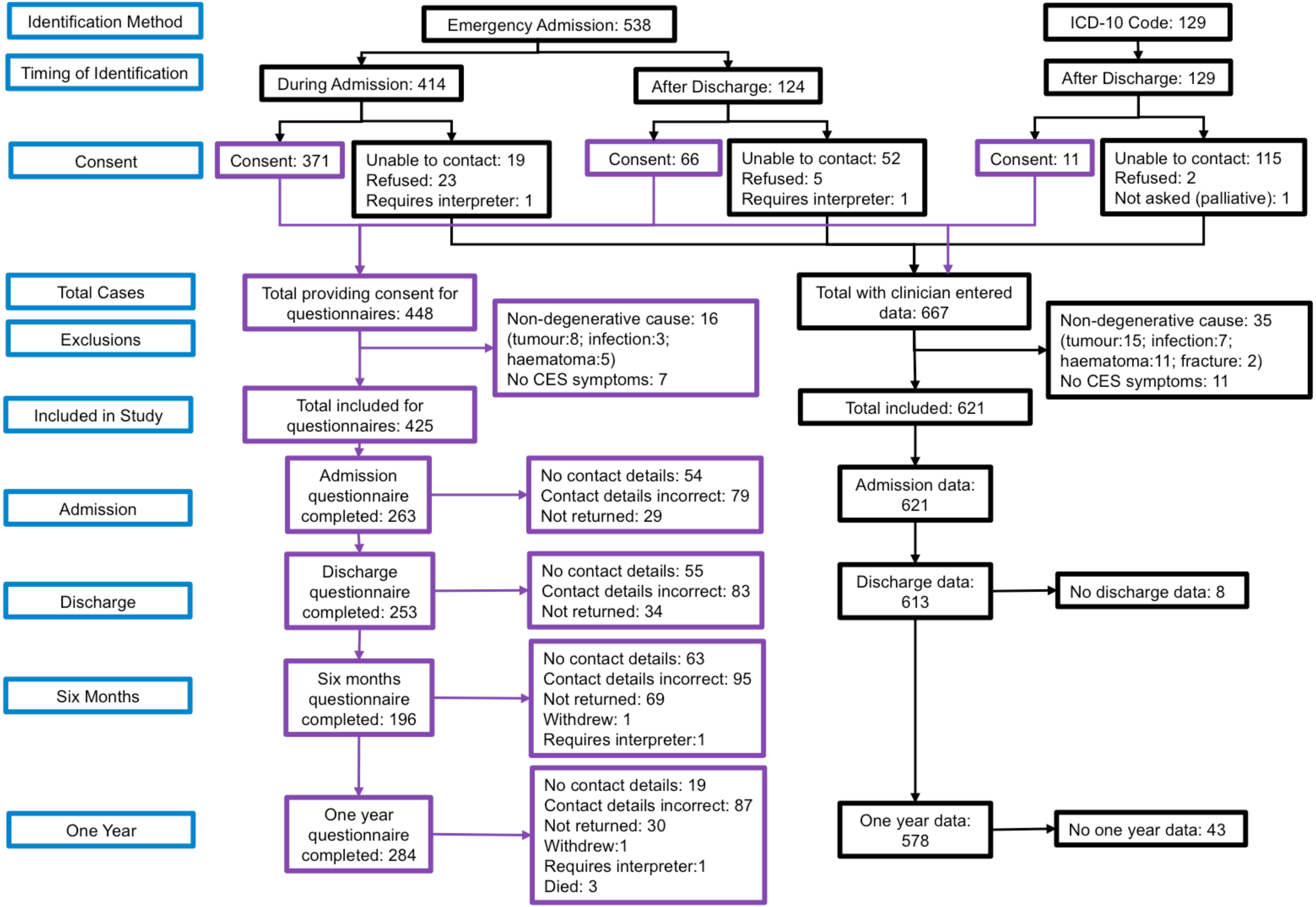
Identification Method
Timing of Identification
Consent
Total Cases
Exclusions
Included in Study
Admission
Discharge
Six Months
One Year
Emergency Admission: 538
ICD-10 Code: 129
During Admission: 414
After Discharge: 124
After Discharge: 129
Consent: 371
Unable to contact: 19
Refused: 23
Requires interpreter: 1
Consent: 66
Unable to contact: 52
Refused: 5
Requires interpreter: 1
Consent: 11
Unable to contact: 115
Refused: 2
Not asked (palliative): 1
Total providing consent for questionnaires: 448
Non-degenerative cause: 16
(tumour:8; infection:3; haematoma:5)
No CES symptoms: 7
Total with clinician entered data: 667
Non-degenerative cause: 35
(tumour:15; infection:7; haematoma:11; fracture: 2)
No CES symptoms: 11
Total included for questionnaires: 425
Total included: 621
Admission questionnaire completed: 263
No contact details: 54
Contact details incorrect: 79
Not returned: 29
Admission data: 621
Discharge questionnaire completed: 253
No contact details: 55
Contact details incorrect: 83
Not returned: 34
Discharge data: 613
No discharge data: 8
Six months questionnaire completed: 196
No contact details: 63
Contact details incorrect: 95
Not returned: 69
Withdrew: 1
Requires interpreter:1
One year questionnaire completed: 284
No contact details: 19
Contact details incorrect: 87
Not returned: 30
Withdrew:1
Requires interpreter:1
Died: 3
One year data: 578
No one year data: 43

Supplementary Figure 2

Clinician Reported Mobility According To Whether Participant Questionnaires Were Completed

Clinician reported mobility is shown at admission and discharge. The top row shows clinician reported mobility for those where both the admission and discharge questionnaires were completed by participants (n=212). The bottom row shows responses where either admission or discharge questionnaires were not completed by participants (n= 409).

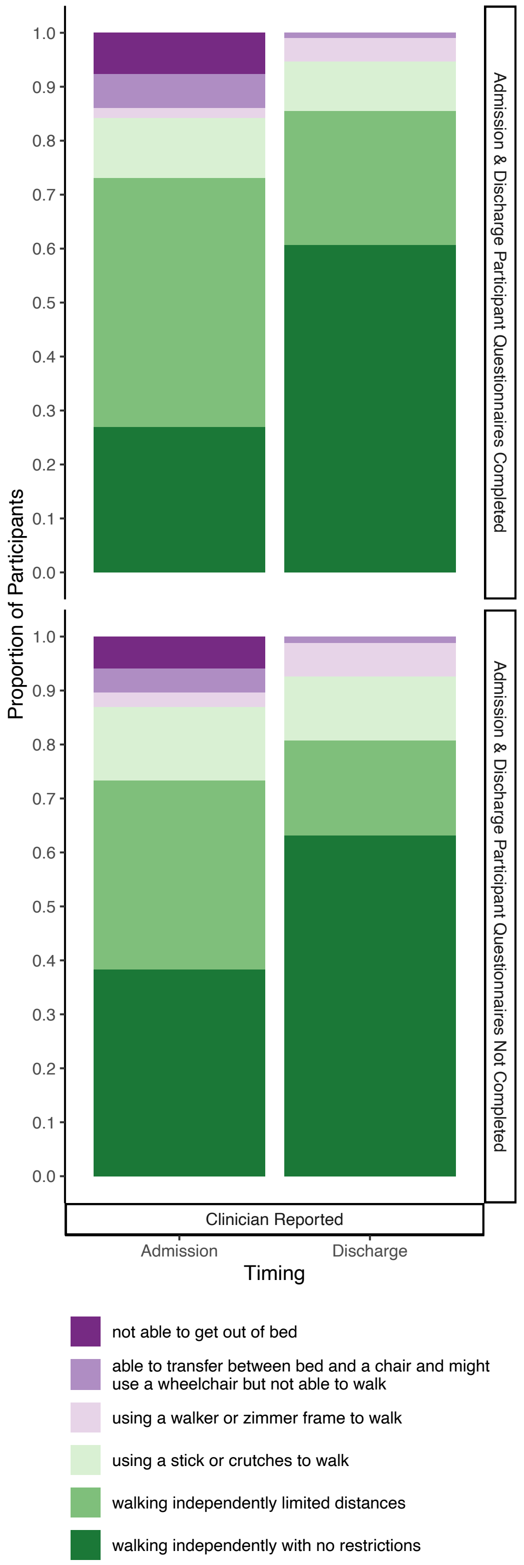

Supplementary Figure 3

Comparison of Clinician and Participant Reported Timing Of Symptom Onset

Time from symptom onset to operation is shown on the x axis. Each bar represents a single participant. Participants are ordered from clinician reported shortest to longest time to operation. Operation timing was clinician reported. Both participants and clinicians reported symptom onset timing. Clinician reported interval from symptom onset to operation is shown in yellow (n=537) and participant reported interval from symptom onset to operation is shown in blue (n=162). If back or leg pain had been present for a long time, both clinicians and participants were asked to report the timing of onset of the problem with bladder function, bowel function, sexual function, numbness, weakness or worsened pain that led to hospital admission. The vertical dotted line marks 48 hours from symptom onset. Only those undergoing surgery as an emergency or on the next day list are shown. Those undergoing surgery on a planned theatre date are not shown (n=41). Intervals longer than 30 days (720 hours) are not shown (n=65). Where intervals in hours are available, these are plotted (n=226). For participants where intervals are only available in days, these have been converted to hours by multiplying by 24, except zero days, which is represented as 12 hours to allow visualisation on the plot.

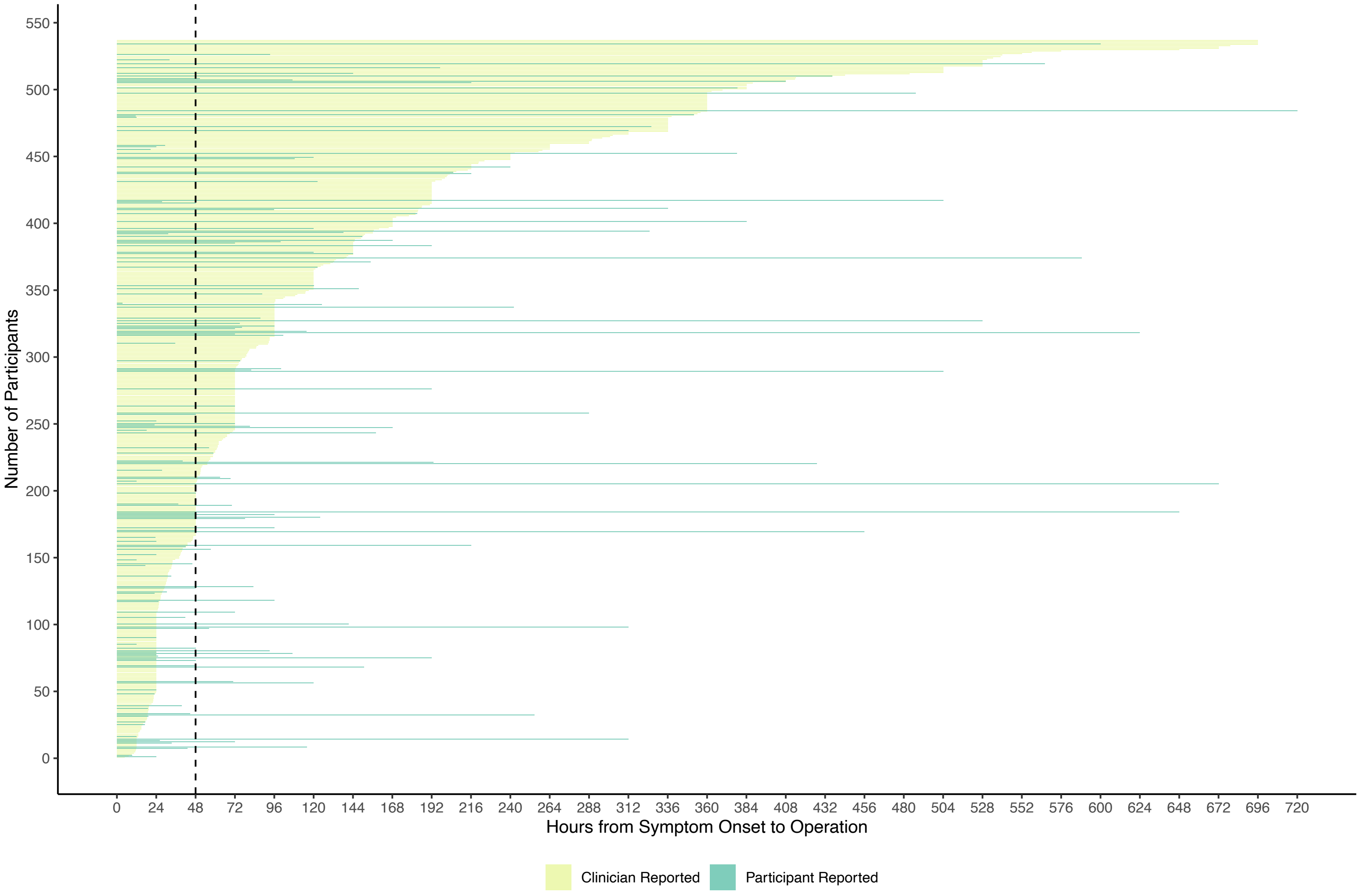

Supplementary Figure 4

Participant Reported Functional Outcomes At One Year

One year post-operative questionnaire responses for the Short Form Incontinence Questionnaire (SFIQ), Neurogenic Bowel Dysfunction (NBD) Score, Arizona Sexual Experiences (ASEX) scale, and the Oswestry Disability Index (ODI). Responses are plotted as percentages of all responses (SFIQ=279, NBD=269 , ASEX=249, ODI=282). All responses are arranged with the least disability at the bottom of the bars (green) and most disability at the top of the bars (purple). SFIQ Q2 is question 2 from the SFIQ and each domain has four possible responses. SFIQ Q3 is question 3 from the SFIQ, with eight possible responses. NBD scores were categorised as per the original description as very minor (0-6), minor (7-9), moderate (10-13), and severe (≥14). Responses for each question of the ASEX are given with 1 being the most favourable (extremely easily / extremely satisfying) and six being least favourable (never / can't). Responses for the ODI are from 0-5 with zero representing no limitation of function and five indicating major functional disability in that domain.

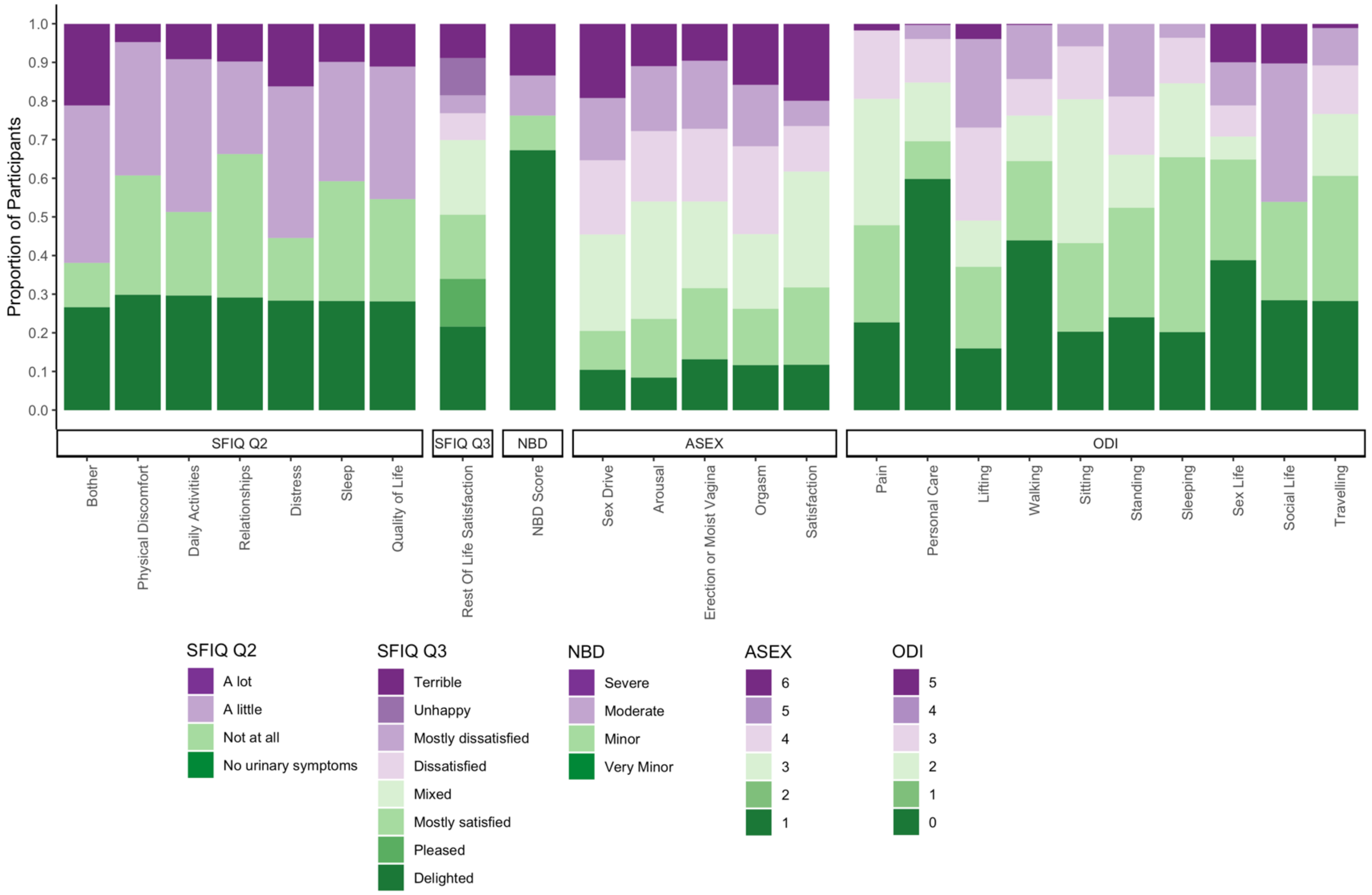

Supplementary Figure 5

Participant Reported Quality of Life at One Year

One year post-operative quality of life questionnaire responses for the 36 Item Short Form Survey (SF-36) domains, Short Form Incontinence Questionnaire (SFIQ), the Oswestry Disability Index (ODI). Responses are converted to percentage scales as per the scoring instructions for each instrument. All questionnaire responses available at one year are plotted (SF-36=270, SFIQ=279, ODI=282). For the SF-36 a higher percentage is representative of higher quality of life. For the SFIQ and ODI, a lower percentage is representative of a higher quality of life.

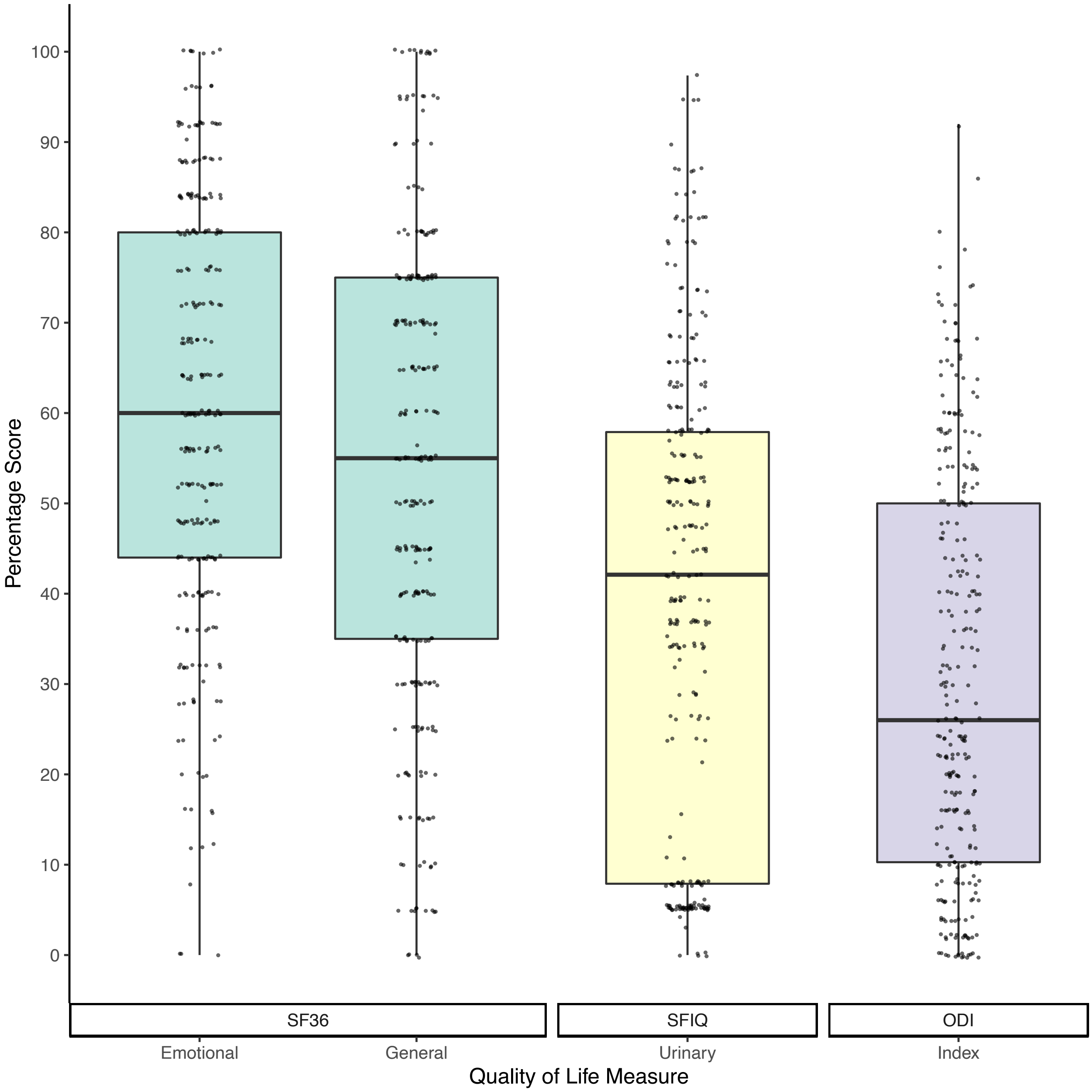

2. Supplementary Tables

Supplementary Table S1

Association of Pre-operative Clinical Findings

Pre-operative clinical findings are positive when either the clinician or participant has reported that symptom at any time pre-operatively, or there were abnormal examination findings. The percentage of participants with the row finding that also has the column finding is calculated. Missing data is removed row and column wise. There were 326 participants where it was unknown if there was sexual dysfunction, and one participant where it was unknown if there were saddle sensory changes (see Table 1).

	**Bladder dysfunction**	**Bowel dysfunction**	**Sexual dysfunction**	**Sciatica**	**Leg Numbness**	**Altered Saddle Sensation**	**Back Pain**	**Leg Weakness**
**Bladder dysfunction**	517 (100%)	223 (43%)	101 (42%)	480 (93%)	398 (77%)	414 (80%)	498 (96%)	316 (61%)
**Bowel dysfunction**	223 (92%)	243 (100%)	76 (60%)	227 (93%)	192 (79%)	212 (87%)	240 (99%)	155 (64%)
**Sexual dysfunction**	101 (88%)	76 (66%)	115 (100%)	113 (98%)	93 (81%)	106 (92%)	110 (96%)	73 (63%)
**Sciatica**	480 (83%)	227 (39%)	113 (39%)	578 (100%)	454 (79%)	471 (82%)	556 (96%)	348 (60%)
**Leg numbness**	398 (83%)	192 (40%)	93 (39%)	454 (94%)	481 (100%)	411 (86%)	464 (96%)	318 (66%)
**Altered Saddle Sensation**	414 (82%)	212 (42%)	106 (43%)	471 (94%)	411 (82%)	502 (100%)	484 (96%)	301 (60%)
**Back Pain**	498 (83%)	240 (40%)	110 (38%)	556 (93%)	464 (78%)	484 (81%)	598 (100%)	364 (61%)
**Leg Weakness**	316 (84%)	155 (41%)	73 (42%)	348 (93%)	318 (85%)	301 (80%)	364 (97%)	376 (100%)

Supplementary Table S2

Bilateral Operative Approaches with CSF Visibility on MRI

Operative approaches are displayed by whether or not CSF was visible at the most affected level on axial T2 MRI. CSF visibility is used as a measure of the amount of compression seen on MRI. Sequestrectomy refers to removal of the sequestered disc fragment only without removal of disc material from the disc space. For interspinous, muscle strip, and discectomy, a single bilateral category is compared against all other categories. For laminectomy, bilateral laminectomy and bilateral laminotomy are combined and compared against all other categories. Missing refers to missing data. (OR: odds ratios, 95% CI: 95% confidence intervals; CSF: cerebrospinal fluid, MRI: magnetic resonance imaging).

		**Visibility of CSF**		**Bilateral vs Unilateral**	
	**Total**	**No CSF visible**	**Some CSF visible**	**OR**	**95% CI**
Interspinous					
Interspinous	*360 (59%)*	*193 (67%)*	*166 (51%)*	*1.9*	*1.4-2.7*
Unilateral	253 (41%)	94 (32%)	157 (49%)		
Missing	8	11			
Muscle Strip					
Bilateral	*332 (54%)*	*186 (65%)*	*145 (45%)*	*2.3*	*1.6-3.2*
Unilateral	280 (46%)	100 (35%)	178 (55%)		
Missing	9	12			
Laminectomy					
Bilateral laminectomy	*241 (39%)*	*132 (46%)*	*108 (33%)*	*2.1*	*1.5-2.9*
Bilateral laminotomy	*78 (13%)*	*45 (16%)*	*33 (10%)*		
Unilateral laminectomy	80 (13%)	29 (10%)	50 (15%)		
Unilateral laminotomy	176 (28%)	64 (22%)	111 (34%)		
No lamina removed	44 (7%)	20 (7%)	24 (7%)		
Missing	2	5			
Discectomy					
Bilateral	*200 (32%)*	*107 (37%)*	*93 (29%)*	1.5	1.0-2.1
Unilateral	301 (49%)	120 (41%)	178 (55%)		
Sequestrectomy	71 (11%)	36 (12%)	35 (11%)		
None	47 (8%)	27 (9%)	20 (6%)		
Missing	2	5			

Supplementary Table S3

Healthcare Professionals Consulted

Healthcare professionals consulted for the symptoms or sequalae of CES up to one year post-operatively. Multiple healthcare professionals could be recorded. Appointments with spinal surgeons or spinal specialist nurses were not included, unless the specialist nurse was also recorded as providing a rehabilitation service. At one year, clinicians may not have had information about appointments occurring outside their hospital. Continence services included urology, gastrointestinal medicine, general surgery, gynaecology, or specialist continence service appointments. Rehabilitation includes neuro-rehabilitation and spinal injuries services. Other healthcare professionals included pain specialists, orthotists, neurologists, general practitioners, rheumatologists, chiropractors, osteopaths, acupuncturists, psychologists, psychiatrists, counsellors, and hypnotists.

	**Clinician Reported**			**Participant Reported**
	**Seen during Admission**	**Referred to at Discharge**	**Seen during one year follow up**	**Seen during one year follow up**
	n=610	n=608	n=490	n=280
No other healthcare professionals seen	21 (3%)	173 (28%)	170 (35%)	95 (34%)
**Healthcare Professionals**				
Physiotherapy	572 (94%)	357 (59%)	228 (47%)	133 (48%)
Occupational Therapy	195 (32%)	58 (10%)	14 (3%)	33 (12%)
Continence	29 (5%)	79 (13%)	72 (15%)	54 (19%)
Rehabilitation	15 (2%)	49 (8%)	30 (6%)	11 (4%)
Other	21 (3%)	13 (2%)	62 (13%)	44 (16%)

4. List of Collaborators

First Name	Middle Names	Last Name	Affiliation
Mohamed		Abdelsadg	Ninewells Hospital, Dundee
Motaz	M S	Abulaila	Royal Hallamshire Hospital, Sheffield
Usman		Ahmed	The Royal Orthopaedic Hospital, Birmingham
Qasim		Ajmi	Royal Preston Hospital, Preston
Rafid		Al-Mahfoudh	University Hospitals Sussex, Sussex
Chadi		Ali	Sunderland Royal Hospital, Sunderland
Meriem		Amarouche	St George's Hospital, London
Amin		Andalib	Hull Royal Infirmary, Hull
Mohit		Arora	Aberdeen Royal Infirmary, Aberdeen
Mukul		Arora	University Hospital Southampton, Southampton
Mariam		Awan	Charing Cross Hospital, London
Afsand		Baig Mirza	King's College Hospital, London
Antony		Bateman	Royal Derby Hospital, Derby
Iwan		Bennett	Charing Cross Hospital, London
Imran		Bhatti	University Hospital of Wales, Cardiff
Peter		Bodkin	Aberdeen Royal Infirmary, Aberdeen
Lalasa		Bommireddy	Royal Derby Hospital, Derby
George		Bonanos	James Cook University Hospital, Middlesborough
Anouk		Borg	The National Hospital for Neurology and Neurosurgery, Queen Square, London
Alexandros		Boukas	John Radcliffe Hospital, Oxford
James		Bourne	Royal Preston Hospital, Preston
Rachael		Brennan	Salford Royal Hospital, Manchester
Jennifer		Brown	Queen Elizabeth University Hospital, Glasgow
Katie		Brown	Department of Clinical Neurosciences, Edinburgh
Oliver		Burton	University Hospital Coventry, Coventry
Christopher		Busby	Royal Derby Hospital, Derby
Neil		Chiverton	Northern General Hospital, Sheffield
Simon		Clark	The Walton Centre, Liverpool
Phillip	C	Copley	Department of Clinical Neurosciences, Edinburgh
Simon		Cudlip	John Radcliffe Hospital, Oxford
Yan		Cunningham	Sunderland Royal Hospital, Sunderland
Ronan		Dardis	University Hospital Coventry, Coventry
Stacey		Darwish	Royal Victoria Hospital, Belfast
Benjamin		Davies	Addenbrooke's Hospital, Cambridge
Andreas	K	Demetriades	Department of Clinical Neurosciences, Edinburgh
Saurabh		Deore	Norfolk and Norwich University Hospital, Norfolk
Chris		Derham	The General Infirmary at Leeds, Leeds
Muhammad		Dherijha	Salford Royal Hospital, Manchester
Gareth		Dobson	James Cook University Hospital, Middlesborough
James		Duncan	Nuffield Orthopaedic Centre, Oxford
Andrew		Durnford	University Hospital Southampton, Southampton
Alexander	Z E	Durst	Norfolk and Norwich University Hospital, Norfolk
Edward	W	Dyson	Charing Cross Hospital, London
Niall		Eames	Royal Victoria Hospital, Belfast
Ellie		Edlmann	Derriford Hospital, Plymouth
Andrew		Edwards-Bailey	Salford Royal Hospital, Manchester
Anne		Elserius	University Hospital Coventry, Coventry
Becca		Elson	Department of Clinical Neurosciences, Edinburgh
Mohammed		Fadelalla	Ninewells Hospital, Dundee
Daniel	M	Fountain	Salford Royal Hospital, Manchester
Adrian		Gardner	The Royal Orthopaedic Hospital, Birmingham
Arnab		Ghosh	Essex Neurological Centre, Romford

James	R	Gill	Norfolk and Norwich University Hospital, Norfolk
Stella	A	Glasmacher	Department of Clinical Neurosciences, Edinburgh
Robin		Gordon	Royal Victoria Hospital, Belfast
Gordan		Grahovac	King's College Hospital, London
Rebecca		Grenfell	University Hospital Coventry, Coventry
Awais		Habeebullah	The Royal Orthopaedic Hospital, Birmingham
Nikolaos		Haliasos	Essex Neurological Centre, Romford
Tim		Hammett	Royal Derby Hospital, Derby
Cathal	J	Hannan	The Walton Centre, Liverpool
Ciaran	S	Hill	Essex Neurological Centre, Romford
Ingrid		Hoeritzauer	Department of Clinical Neurosciences, Edinburgh
David		Holmes	Charing Cross Hospital, London
Kismet		Hossain-Ibrahim	Ninewells Hospital, Dundee
Laura		Hughes	Southmead Hospital, Bristol
Muhammad		Hussain	Hull Royal Infirmary, Hull
Shakir		Hussain	The Royal Orthopaedic Hospital, Birmingham
Ramez		Ibrahim	Royal Hallamshire Hospital, Sheffield
Aimun	A B	Jamjoom	Aberdeen Royal Infirmary
Bethan		John	The Walton Centre, Liverpool
Shabin		Joshi	University Hospital Coventry, Coventry
Josephine		Jung	King's College Hospital, London
Oliver		Kennion	Department of Clinical Neurosciences, Edinburgh
Muhammad		Khan	Addenbrooke's Hospital, Cambridge
Adriana		Klejnotowska	Department of Clinical Neurosciences, Edinburgh
Ashwin		Kumaria	Royal Derby Hospital, Derby
Roberta		LaCava	Royal Sussex County Hospital, Brighton
Simon		Lammy	Queen Elizabeth University Hospital, Glasgow
Alistair		Lawrence	Addenbrooke's Hospital, Cambridge
Matthew		Lea	Royal Preston Hospital, Preston
Andraay	H C	Leung	Queen Elizabeth University Hospital, Glasgow
Ignatius		Liew	The Walton Centre, Liverpool
Weisang		Luo	The Walton Centre, Liverpool
Oscar		MacCormac	Charing Cross Hospital, London
James		Manfield	Royal Preston Hospital, Preston
Richard		Mannion	Addenbrooke's Hospital, Cambridge
Joseph		Merola	University Hospital of Wales, Cardiff
Pranav		Mishra	Hull Royal Infirmary, Hull
Khalid	A	Mohmoud	James Cook University Hospital, Middlesborough
Richard		Moon	Southmead Hospital, Bristol
Rory		Morrison	British Orthopaedic Trainee Association
Odhran		Murray	Queen Elizabeth University Hospital, Glasgow
Ali		Nader-Sepahi	University Hospital Southampton, Southampton
Colin		Nnandi	Nuffield Orthopaedic Centre, Oxford
Anand		Pandit	The National Hospital for Neurology and Neurosurgery, Queen Square, London
Nitin		Patel	Southmead Hospital, Bristol
Anita		Philip	Southmead Hospital, Bristol
Michael	T C	Poon	Department of Clinical Neurosciences, Edinburgh
Kuskoor	S M	Prasad	James Cook University Hospital, Middlesborough
Savva		Pronin	Department of Clinical Neurosciences, Edinburgh
Shyam		Pujara	Hull Royal Infirmary, Hull
Balaji		Purushothaman	Sunderland Royal Hospital, Sunderland
Kapil		Rajwani	Royal Sussex County Hospital, Brighton
Fahid	T	Rasul	Essex Neurological Centre, Romford
Holly		Roy	Derriford Hospital, Plymouth
Ahmed-Ramadan		Sadek	University Hospital Southampton, Southampton
Moritz		Schramm	The General Infirmary at Leeds, Leeds

Gabrielle		Scicluna	Queen Elizabeth University Hospital, Glasgow
Philip	J	Sell	University Hospitals of Leicester NHS Trust, Leicester
Roozbeh		Shafafy	Royal Sussex County Hospital, Brighton
Himanshu		Sharma	Derriford Hospital, Plymouth
Asim		Sheikh	The General Infirmary at Leeds, Leeds
Vinothan		Sivasubramaniam	Royal Sussex County Hospital, Brighton
Agbolahan		Sofela	Derriford Hospital, Plymouth
George		Spink	Hull Royal Infirmary, Hull
Nisaharan		Srikandarajah	The Walton Centre, Liverpool
Patrick	F X	Statham	Department of Clinical Neurosciences, Edinburgh
Stuart		Stokes	Royal Hallamshire Hospital, Sheffield
Euan		Strachan	Charing Cross Hospital, London
Chrishan		Thakar	Nuffield Orthopaedic Centre, Oxford
Gopiga		Thanabalasundaram	University Hospital Coventry, Coventry
Paul		Thorpe	Musgrove Park Hospital, Taunton
Christian		Ulbricht	Charing Cross Hospital, London
Anna		Watts	Northern General Hospital, Sheffield
Alison		Whitcher	Musgrove Park Hospital, Taunton
David		White	Royal Derby Hospital, Derby
Kathrin		Whitehouse	Southmead Hospital, Bristol
Martin		Wilby	The Walton Centre, Liverpool
Julie		Woodfield	Department of Clinical Neurosciences, Edinburgh
Ardalan		Zolnourian	University Hospital Southampton, Southampton

5. Participant Information Sheet

Understanding Cauda Equina Syndrome

UNDERSTANDING CAUDA EQUINA SYNDROME (UCES)

What is the purpose of this study?

Cauda equina syndrome occurs when the cauda equina nerve roots in the lower spine are compressed. This can cause a range of symptoms such as pain, leg weakness, leg numbness, and bladder, bowel, or sexual dysfunction. This study aims to:

- describe which symptoms people with cauda equina syndrome experience,
- look at the way that people with cauda equina syndrome are currently managed, and
- find out what happens to people after they are discharged from hospital.

This will help us plan how patients with cauda equina syndrome are investigated and managed in the future.

Why have I been invited to take part?

You have been asked to take part because you were admitted to hospital with compression of the cauda equina nerve roots.

What will happen if I take part?

One of the team caring for you will discuss the project with you and fill in the consent form for the study with you. After this, information about your symptoms, investigation and management from your medical notes will be entered anonymously into a study database by the team that are treating you. We will also send you questionnaires to complete about your symptoms. We will ask you about how you were at four time points:

1. Just prior to admission to hospital
2. On discharge from hospital
3. Six months after your operation
4. One year after your operation

We expect the questionnaires to take a maximum of 10 minutes each to complete.

Some of the questions will be about your bladder, bowel, and sexual function. If you do not wish to answer these questions you can leave them blank. If you are happy to fill in the questionnaires electronically we will ask for your email address and you will be emailed a personalised link to fill in the questionnaires. Your email address will be stored in an encrypted study database and only the clinical team caring for you will have access to it. Your answers cannot be identified to your email address except by the team looking after you. If you would prefer to fill in the questionnaires on paper or over the telephone we will give you the first two questionnaires in hospital. We will then share your contact details with the central study team at NHS Lothian who will contact you via post or telephone to complete the last two questionnaires. We will also ask for your permission to anonymously link data collected as part of this study to any routinely collected existing records in databases or registries.

What will happen to the results of this study?

The study results will be published in journal articles and presented at conferences and at the local study centres with the aim of improving care for people with cauda equina syndrome in the future. We will also share the results of the study with all the participants using the contact details we have used to send you the questionnaires. You will not be identifiable in any published results.

What are the advantages and disadvantages of taking part?

There are no direct benefits to taking part in this study but the results from this study might help to improve the healthcare of patients in the future. This study will take up to 40 minutes of your time over the course of the year following your admission to hospital.

PARTICIPANT INFORMATION LEAFLET

UCES
Understanding Cauda Equina Syndrome

NHS Lothian

UNDERSTANDING CAUDA EQUINA SYNDROME (UCES)

PARTICIPANT INFORMATION LEAFLET

Do I have to take part?

No, you can decide whether or not you wish to take part. Your decision will not affect the care that your receive. If you decide to take part you are still free to withdraw from the study at any time without giving a reason. If you don't want to carry on with the study after you have signed the consent form, please tell us that you want us to stop sending you the questionnaires. Our contact details are at the bottom of this sheet and on all the questionnaires that we will send to you. If you become unable to make a decision about participating in this study we will not send you any further questionnaires but we will still use the data that you have previously agreed to enter into the study.

Will my study data be kept confidential?

All the information that we collect during the study will be kept confidential. For the majority of participants only anonymous data (which cannot be used to identify you) will be shared outside the NHS organisation that is treating you. If you choose to complete the questionnaires on paper or over the telephone, or if we don't hear back from you then your contact details will be shared with the central study team at NHS Lothian via secure NHS email addresses. The central team will contact you to check if you still wish to take part in the study and how you wish to complete the questionnaires. Your anonymous data will be stored securely for five years and might be used in future ethically approved studies. The anonymous dataset may be processed on computers outside the NHS. To monitor and audit the study to ensure it is being appropriately conducted, responsible representatives from the study sponsor and NHS institutions may access your medical records and data collected during the study.

Who is organising this study?

This study has been organised by the British Neurosurgical Trainee Research Collaborative in conjunction with the British Orthopaedic Trainee Association and the British Association of Spine Surgeons. The study is sponsored by NHS Lothian. All research in the NHS is looked at by an independent group of people called a Research Ethics Committee. A favourable ethical opinion has been obtained from the South East Scotland Research Ethics Committee. NHS management approval at each site at which the study is being carried out has also been given.

Who can I contact about this study?

If you have further questions about the study please discuss these with the team looking after you or your local study contact:

- Dr Ingrid Hoeritzauer, Department of Neurology, NHS Lothian, 0131 537 1000 or Ingrid.Hoeritzaeur@nhslothian.scot.nhs.uk

If you would like to contact the team in charge of the study please contact:

- Miss Julie Woodfield, Department of Neurosurgery, NHS Lothian, 0131 537 1000 or julie.woodfield@nhs.net

If you would like to discuss this study with someone who is independent of the study team and is a clinician within the NHS Lothian Neurosurgery unit you can contact:

- Mr Paul Brennan, Consultant Neurosurgeon, NHS Lothian, 0131 537 2106 or paul.brennan@nhslothian.scot.nhs.uk

If you wish to make a complaint at any stage of the study, please speak to the team looking after you, your local study contact, or the team in charge of the study. If you remain unhappy and wish to complain formally you can do this through the NHS complaints procedure. Please contact:

- Patient Experience Team, 2-4 Waterloo Place, Edinburgh. EH1 3EG, 0131 536 3370 or feedback@nhslothian.scot.nhs.uk

6. Consent Form

UCES
Understanding Cauda Equina Syndrome

NHS Lothian

UNDERSTANDING CAUDA EQUINA SYNDROME (UCES)

Castor ID:	

(Generated when new record created in database)

CONSENT FORM

- I confirm that I have read and understand the participant information leaflet (version 2.0,16/04/18) for the above study. I have had the opportunity to consider the information, ask questions and have had these questions answered satisfactorily. ☐
- I understand that my participation is voluntary and that I am free to withdraw at any time without giving any reason and without my medical care and/or legal rights being affected. ☐
- I give permission for my healthcare team to share anonymised data from my medical records, including my scans. This data will be encrypted and stored within the UK. ☐
- I give permission for my GP to be contacted about my participation in the study. ☐
- I give permission for my contact details (name, email address, postal address, telephone number) to be passed to NHS Lothian for administration of the study. Contact details will be stored securely on NHS password protected computers. ☐
- I agree to my study data being linked to my existing records in databases or registries and for the linked data to be used for research purposes. ☐
- I understand that relevant sections of my medical notes and data collected during the study may be looked at by individuals from regulatory authorities, the sponsor (NHS Lothian), or other NHS health boards to ensure the study is being appropriately conducted. I agree to these individuals accessing my records. ☐
- I agree to my anonymised data being used in future ethically approved studies. Yes ☐ No ☐
- I agree to my anonymised data being processed as part of the study dataset on computers outside the NHS. I understand that my data will be held securely and stored in encrypted files on password protected computers at all times. ☐
- I agree to take part in the above study. ☐

Name of Person Giving Consent	Date	Signature
Name of Person Receiving Consent	Date	Signature

1x original – into Site File; 1x copy – to Participant;

British Neurosurgical Trainee Research Collaborative BNTRC Advancing Research through Collaboration

BOTA British Orthopaedic Trainees Association

BASS British Association of Spine Surgeons

UNDERSTANDING CAUDA EQUINA SYNDROME (UCES)

**Castor ID:**	

(Generated when new record created in database)

CONTACT DETAILS

Name:	
Email Address:	
Postal Address:	
Telephone Number:	

British Neurosurgical Trainee Research Collaborative BNTRC Advancing Research through Collaboration
BOTA British Orthopaedic Trainees Association
BASS British Association of Spine Surgeons

7. Study Protocol

British Neurosurgical Trainee Research Collaborative

BNTRC

Advancing Research through Collaboration

BOTA

British Orthopaedic Trainees Association

BASS

British Association of Spine Surgeons

NHS

Lothian

UCES

Understanding Cauda Equina Syndrome

STUDY PROTOCOL

UNDERSTANDING CAUDA EQUINA SYNDROME (UCES)

Sponsor	NHS Lothian<br>ACCORD<br>The Queen's Medical Research Institute<br>47 Little France Crescent<br>Edinburgh<br>EH16 4TJ
Protocol Authors	Julie Woodfield, Ingrid Hoeritzauer, Aimun Jamjoom
Study Name	Understanding Cauda Equina Syndrome
Joint Chief Investigators	Miss Julie Woodfield<br>Dr Ingrid Hoeritzauer<br>Mr Aimun Jamjoom
Sponsor Reference	AC18017
IRAS Reference	233515
REC Number	18/SS/0047
Project Registration	Protocol published at www.bntrc.org.uk
Version Number and Date	Version 4.0, 11th April 2019

STUDY PROTOCOL

STEERING COMMITTEE

Mr Simon Clark<br><br>Consultant Neurosurgeon<br>The Walton Centre, Liverpool<br><br>simon.clark@thewaltoncentre.nhs.uk	Mr Andreas Demetriades<br><br>Consultant Neurosurgeon<br>NHS Lothian<br><br>A.Demetriades@nhslothian.scot.nhs.uk
Mr Gareth Dobson<br><br>Neurosurgery Specialty Trainee<br>Northern Deanery<br><br>gareth_dobson@talk21.com	Mr Niall Eames<br><br>Consultant Orthopaedic Surgeon<br>Belfast Health and Social Care Trust<br><br>nialleames.spine@btinternet.com
Dr Ingrid Hoeritzaur<br><br>Neurology Specialty Trainee<br>NHS Lothian<br><br>Ingrid.Hoeritzauer@ed.ac.uk	Mr Aimun Jamjoom<br><br>Neurosurgery Specialty Trainee<br>NHS Lothian<br><br>aabjamjoom@gmail.com
Mr Andraay Leung<br><br>Orthopaedic Specialty Trainee<br>NHS Greater Glasgow and Clyde<br><br>andraayleung@gmail.com	Dr Michael Poon<br><br>Neurosurgery Specialty Trainee<br>NHS Lothian<br><br>michael.poontc@gmail.com
Mr Manjunath Prasad<br><br>Consultant Neurosurgeon<br>South Tees Hospitals NHS Foundation Trust<br><br>manjunath.prasad@stees.nhs.uk	Mr Savva Pronin<br><br>Medical Student<br>University of Edinburgh, Edinburgh<br><br>s1206328@sms.ed.ac.uk
Mr Phil Sell<br><br>Consultant Orthopaedic Surgeon<br>Nottingham University Hospitals NHS Trust<br><br>philip.sell@nuh.nhs.uk	Mr Nish Srikandarajah<br><br>Neurosurgery Specialty Trainee<br>The Walton Centre, Liverpool<br><br>nish.sri@doctors.org.uk
Mr Patrick Statham<br><br>Consultant Neurosurgeon<br>Western General Hospital, Edinburgh<br><br>Patrick.Statham@nhslothian.scot nhs.uk	Miss Stacey Thomson<br><br>Orthopaedic Specialty Trainee<br>Belfast Health and Social Care Trust<br><br>stacey.thomson@belfasttrust.hscni.net
Mr Martin Wilby<br><br>Consultant Neurosurgeon<br>The Walton Centre, Liverpool<br><br>martin.wilby@thewaltoncentre.nhs.uk	Miss Julie Woodfield<br><br>Neurosurgery Specialty Trainee<br>Western General Hospital, Edinburgh<br><br>julie.woodfield@ed.ac.uk

PARTICIPATING SITES

Site	Local Investigator
**Scotland**	
NHS Lothian	Philip Copley
NHS Grampian	Aimun Jamjoom
NHS Greater Glasgow & Clyde	Simon Lammy
NHS Tayside	Kismet Hossain-Ibrahim
**Northern Ireland**	
Belfast Health and Social Care Trust	Niall Eames
**England**	
The Walton Centre NHS Foundation Trust	Nish Srikandarajah
Royal Liverpool and Broadgreen University Hospitals NHS Trust	Anil Dhadwal
The Newcastle Upon Tyne Hospitals NHS Foundation Trust	Ashan Jayasekera
South Tees Hospitals NHS Foundation Trust	Manjunath Prasad
Lancashire Teaching Hospitals NHS Foundation Trust	Jim Bourne
City Hospitals Sunderland NHS Foundation Trust	Balaji Purushothaman
Salford Royal NHS Foundation Trust	Muhammad Dherija
Hull and East Yorkshire Hospitals NHS Trust	Amin Andalib
Leeds Teaching Hospitals NHS Trust	Asim Sheikh
Sheffield Teaching Hospitals NHS Foundation Trust	Stuart Stokes
Nottingham University Hospitals NHS Trust	Phil Sell
Derby Hospitals NHS Foundation Trust	Tim Hammet
University Hospital Birmingham NHS Foundation Trust	Navin Furtado
The Royal Orthopaedic Hospital NHS Foundation Trust	Adrian Gardner
University Hospitals Coventry and Warwickshire NHS Trust	Ronan Dardis
University Hospital Southampton NHS Foundation Trust	Ardalan Zolnourian
Cambridge University Hospitals NHS Foundation Trust	Richard Mannion
Norfolk and Norwich University Hospitals NHS Foundation Trust	Alexander Durst
East Kent Hospitals University NHS Foundation Trust	Abdulrahman Abed
Oxford University Hospitals NHS Trust	Alexandros Boukas
North Bristol NHS Trust	Richard Moon
Plymouth Hospitals NHS Trust	Ellie Edlmann
Royal Devon and Exeter NHS Foundation Trust	Mike Hutton
Taunton and Somerset NHS Foundation Trust	Paul Thorpe
Buckinghamshire Healthcare NHS Trust	Abayomi Animashawun
Milton Keynes Hospital NHS Trust	Andrew James
Ipswich Hospital NHS Trust	David Cumming
The Princess Alexandra Hospital NHS Trust	Ramal Ganasekaran
Barts Health NHS Trust	Thomas Doke
Barking Havering and Redbridge University Hospitals NHS Trust	Fahid Rasul
King's College Hospital NHS Foundation Trust	Josephine Jung
Brighton and Sussex University Hospitals NHS Trust	Rafid Al-Mahfoudh
St George's Healthcare NHS Foundation Trust	Meriem Amarouche
University College London Hospitals NHS Foundation Trust	Anand Pandit
Imperial College Healthcare NHS Trust	Edward Dyson
**Wales**	
Cardiff and Vale University Health Board	Aziz Ahmad

CONTENTS

1 INTRODUCTION 7
1.1 BACKGROUND 7
1.2 CURRENT STANDARDS OF CARE 7
2 OBJECTIVES 8
3 STUDY DESIGN 8
4 STUDY POPULATION 9
4.1 NUMBER OF PARTICIPANTS 9
4.2 INCLUSION CRITERIA 9
4.3 EXCLUSION CRITERIA 9
4.4 CO-ENROLMENT 9
5 PARTICIPANT SELECTION AND ENROLMENT 10
5.1 IDENTIFYING PARTICIPANTS 10
5.2 CONSENTING PARTICIPANTS 10
5.3 WITHDRAWAL OF STUDY PARTICIPANTS 10
6 DATA COLLECTION 10
7 STATISTICS AND DATA ANALYSIS 12
7.1 SAMPLE SIZE CALCULATION 12
7.2 PROPOSED ANALYSES 12
8 OVERSIGHT ARRANGEMENTS 13
8.1 INSPECTION OF RECORDS 13
9 GOOD CLINICAL PRACTICE 13
9.1 ETHICAL CONDUCT 13
9.2 INVESTIGATOR RESPONSIBILITIES 13
10 STUDY CONDUCT RESPONSIBILITIES 14
10.1 PROTOCOL AMENDMENTS 14
10.2 MANAGEMENT OF PROTOCOL NON COMPLIANCE 14
10.3 SERIOUS BREACH REQUIREMENTS 14
10.4 STUDY RECORD RETENTION 15
10.5 END OF STUDY 15
10.6 INSURANCE AND INDEMNITY 15
11 REPORTING, PUBLICATIONS AND NOTIFICATION OF RESULTS 15
11.1 AUTHORSHIP POLICY 15
11.2 PUBLICATION 15
11.3 PEER REVIEW 15
12 REFERENCES 16

ABBREVIATIONS

ACCORD	Academic and Clinical Central Office for Research & Development - Joint office for The University of Edinburgh and Lothian Health Board
BASS	British Association of Spine Surgeons
BNTRC	British Neurosurgical Trainee Research Collaborative
BOTA	British Orthopaedic Trainees Association
CES	Cauda Equina Syndrome
CESI	Cauda equina syndrome - incomplete
CESR	Cauda equina syndrome - retention
CESS	Cauda equina syndrome - suspected
CHI	Community Health Index
CI	Chief Investigator
CRF	Case Report Form
DGH	District General Hospital
DRE	Digital Rectal Examination
EU	European Union
GCP	Good Clinical Practice
ICMJE	International Committee of Medical Journals Editors
ICD-10	International Statistical Classification of Diseases and Related Health Problems, 10th version
ICH	International Conference on Harmonisation
ISF	Investigator Site File
MRI	Magnetic Resonance Imaging
NHS	National Health Service
PACS	Picture Archiving and Communications System
PI	Principal Investigator
QA	Quality Assurance
REC	Research Ethics Committee
SOP	Standard Operating Procedure
UCES	Understanding Cauda Equina Syndrome
UK	United Kingdom

SUMMARY

Cauda equina syndrome is a potentially devastating condition caused by compression of the cauda equina nerve roots. This can result in bowel, bladder and sexual dysfunction plus lower limb weakness, numbness, and pain. Cauda equina syndrome occurs infrequently but has serious potential morbidity and medico-legal consequences. This study aims to identify and describe the presentation and management of patients with cauda equina syndrome in the United Kingdom using trainee research collaborative networks. This will provide accurate incidence figures, establish current clinical practice, allow assessment of the adherence to national published standards of care, and determine patient outcomes from this condition. Accurate, up to date information about the presentation, management, and outcome of patients with cauda equina syndrome will inform standards of service design and delivery for this important but infrequent condition and help to identify future research priorities.

1 INTRODUCTION

1.1 BACKGROUND

Cauda equina syndrome (CES) is a rare but potentially devastating condition caused by compression of the cauda equina nerve roots. This most commonly occurs due to a prolapsed intervertebral disc. The clinical syndrome includes any of bilateral sciatica, saddle anaesthesia, bladder, bowel, and sexual dysfunction.[1-3] The disabling nature of these symptoms causes significant medical and social morbidity and high health and social care costs. In addition, litigation related to the management of CES leads to significant medicolegal workload and costs.[1, 4, 5]

Due to the consequences of CES for patients and society, several groups have issued clinical guidance or standards of care for CES.[1, 6-8] However, the evidence base for current clinical guidance consists of small retrospective single centre case series.[1, 9, 10] Even systematic reviews of outcomes in CES have included relatively few patients, with the largest including 464 patients.[9, 11] Lack of a clear definition of CES has hampered comparative analysis of historic studies, and different interpretations of the available evidence have been offered.[10, 12] A diagnosis of CES encompasses patients presenting with any of urinary hesitancy, complete painless urinary retention, perineal or perianal numbness, faecal incontinence, sexual function disturbance, or bilateral sciatica, but patients may also experience lower limb weakness, numbness, or unilateral sciatica.[2, 3, 13] Accurate division of CES into subtypes of suspected (CESS), incomplete (CESI), and with retention (CESR) can help to clarify understanding of outcome studies and develop care standards appropriate to the presentation,[1, 14] but the current evidence is based on a small number of patients.

Retrospective case series in the UK have identified approximately 15-31 patients per year per specialist neurosurgical or spinal centre with confirmed CES.[3, 13, 15, 16] Estimates of the incidence of CES range from 1-7 per 100,000 population.[17, 18] In 2010-2011 in England, 981 surgical decompressions were performed for CES according to hospital episode statistics,[19] and the population was estimated at 52,234,000,[20] giving an incidence of 1.9 per 100,000 for operated CES. Although CES is not common, there may still be over 1000 patients managed for CES in the UK per year. Accurate data on the presentation and management of these patients would establish current management and adherence to and feasibility of care quality statements as well as potentially informing the revision of guidance based on accurate and current data.

The British Neurosurgical Trainee Research Collaborative (BNTRC) has previously successfully used a network of neurosurgical trainees across the United Kingdom (UK) and Ireland to identify cases via local tertiary referral systems in conditions such as chronic subdural haematoma.[21] As CES is managed in the UK and Ireland by specialist spinal services, similar case ascertainment via specialist referral systems to neurosurgical, orthopaedic, or joint spinal services provides a method of accurately identifying patients with CES during hospital admission. This would allow prospective identification and assessment of large numbers of patients across the UK and Ireland over a defined time period, and lead to the largest prospective observational cohort study of CES.

We propose to carry out the first national cohort study of the presentation and management of CES in the UK and Ireland and establish the largest prospective series of patients with CES. This will provide data on CES incidence, epidemiology, presentation, management, and outcomes. This will inform the development of clinical guidance and identify areas for future research in this condition.

1.2 CURRENT STANDARDS OF CARE

The British Association of Spine Surgeons (BASS) standards of care for suspected and confirmed compressive CES issued in 2015 include the following main statements.[6]

- Imaging, usually Magnetic Resonance Imaging (MRI), should be undertaken as an emergency.
- Decompressive surgery should be undertaken at the earliest opportunity, taking into consideration the duration of pre-existing symptoms and the potential for increased morbidity while operating in the small hours.
- Patients should be counselled that the aim of surgery is to preserve that function present at the time of surgery.

The Society of British Neurological Surgeons Care Quality Statement issued in October 2015 includes the following statements relevant to the management of patients with CES.[8]

- Appropriate and timely management of acute neurosurgical disorders should always take priority over less urgent elective cases.
- All patients should be appropriately investigated in a timely manner by secondary care prior to referral.
- It is not appropriate that neurosurgical staffs are called upon to act as a scanning service and a diagnosis should be reached prior to referral.
- Out of hours MRI scanning for emergency or urgent clinical problems should be considered to be routine practice prior to referral in order to prevent the needless and potentially harmful transport of patients for diagnostic imaging.
- Trusts must make remote access to imaging instantly available to Consultant Neurosurgeons and Neuroradiologists.

A proposal for evidence based guidance for the management of CES in the UK included the following suggestions.[1]

- Accurate history taking of relevant symptoms should be undertaken, including bilateral radicular pain, dermatomal sensory loss, myotomal weakness, and any change in bladder or bowel function.
- Careful examination should be performed to identify objective signs of bilateral radiculopathy, including reflex changes, dermatomal sensory loss, and myotomal weakness.
- Examination of the perineum and digital rectal examination (DRE) should be performed.
- Ideally the MRI should be performed at the District General Hospital within one hour of the request.
- Patients with CESI or CESR with uncertain, early, or preserved cauda equina function should have MRI and surgery as an emergency by day or night
- Patients with CESS can have MRI imaging the following morning with consideration of operating on the same day.
- Patients with prolonged CESR, who are uncertain CES, or who have no preservation of cauda equina function clinically can be operated on the following morning acute list.
- Surgery should be carried out by a consultant spinal surgeon or as a minimum a trainee directly supervised by a consultant

2 OBJECTIVES

This prospective observational cohort study aims to:

- Identify the number of cases of CES in the UK in all collaborating centres
- Describe the presenting symptoms and signs in patients with CES
- Describe the pathways of presentation to specialist spinal services for patients with CES in the UK and Ireland
- Describe the type and timing of investigation of patients with CES
- Describe the medical and surgical management of CES
- Compare current practice to standards of care for CES
- Describe clinical outcomes for patients with CES using validated patient reported outcome measures, stratified by presentation, investigations, and management
- Demonstrate the ability of neurosurgical and orthopaedic surgical trainee networks to collaborate successfully on a prospective cohort study

3 STUDY DESIGN

Understanding Cauda Equina Syndrome (UCES) is a prospective cohort study of patients with confirmed CES managed at specialist spinal centres in the UK.

Cases will be identified by neurosurgical or orthopaedic trainees in each specialist centre through daily screening of tertiary referrals and admissions to specialist spinal services. All patients managed as CES by the treating team will be included in this audit. CES will be divided into CES suspected (CESS), CES incomplete (CESI), and CES retention (CESR).

Data regarding timing and type of symptom onset, referral, investigation, management, and outcome will be recorded anonymously on a secure database by the local trainee investigator during the patient's hospital admission and after discharge. Patient consent will be sought for the use of their data and patients will be asked to complete patient reported outcome measures representing their condition before surgery and up to one year after surgery. Their MRI scans at presentation will also be collected. This data will be compared with care quality statements and published outcome data for CES.

The study will recruit for one year. Cases will be identified from admissions to spinal units between 1st June 2018 until 31st May 2019. The last one year follow up assessments will be sent to participants on 1st June 2020.

This is an observational study. No changes to routine patient care will occur during this study.

4 STUDY POPULATION

4.1 NUMBER OF PARTICIPANTS

This study aims to establish a case series of patients with CES. We expect approximately 20 patients per spinal centre per year depending on the population served, and a total of approximately 600-1000 patients in one year across the UK.

4.2 INCLUSION CRITERIA

For inclusion in this study, the patient must:

- be over 18 years old;
- be admitted to a specialist spinal service in the UK between 1st June 2018 and 31st May 2019;
- have capacity to provide informed consent for participation in this study; and
- have a diagnosis of clinical CES and structural compression of the cauda equina on imaging as determined by the treating clinician.
    - Clinical CES includes any of: altered saddle sensation; bladder dysfunction; bowel dysfunction; sexual dysfunction; or bilateral sciatica. This should be associated with radiological compression of the cauda equina. The cauda equina compression can be due to any cause, including, but not limited to, disc, tumour, infection, etc.

There is no upper age limit as we aim to establish the demographics of those presenting with CES. Participating centres are listed at the beginning of this protocol.

4.3 EXCLUSION CRITERIA

- Patients under 18 years old
- Patients undergoing emergent decompression for unilateral motor or sensory symptoms (eg foot drop), without clinical evidence of CES
- Patients referred with suspected CES where the diagnosis is not confirmed, for example:
    - Patients with the clinical symptoms and signs of CES without radiological evidence of cauda equina compression
- Patients not admitted to participating spinal centres in the UK
- Patients admitted to a participating spinal centre before 1st June 2018 or after 31st May 2019
- Patients who are unable to provide informed consent for participation in this study

4.4 CO-ENROLMENT

Co-enrolment in any other ongoing studies will be allowed.

5 PARTICIPANT SELECTION AND ENROLMENT

5.1 IDENTIFYING PARTICIPANTS

All neurosurgical and orthopaedic units managing patients with CES in the UK will be invited to participate. All patients admitted with radiologically confirmed CES will be identified by the lead local investigator via the screening of referral and admission systems at their spinal unit. All lead local investigators must be members of the clinical team caring for patients with CES in their unit.

Capture-recapture methods will be used to ensure complete case ascertainment. In December 2018, June 2019, and December 2019 all local investigators will check their case ascertainment by asking their local coding departments for all discharges coded as CES using the diagnostic code ICD-10 G83.4. Any additional patients identified through this method that meet the inclusion criteria will be contacted by a member of the clinical team caring for the patient and invited to participate.

5.2 CONSENTING PARTICIPANTS

Once patients have been identified as being eligible to participate in the study, they will be asked by a member of their clinical team whether they would be willing to receive further information about the study. For the majority of patients this will occur during their admission to the spinal unit, and the approach will be made by a member of ward medical or nursing staff. Once verbal consent has been gained to give further information about the study, patients will be provided with the information leaflet for the study. Patients who indicate that they are happy to have further discussions regarding the study will be visited in hospital by a member of their clinical team to complete the written consent process. The person undertaking written consent will be adequately trained to do so, and have a good knowledge of the study protocol, aims, and processes.

Decompression surgery for CES takes place as an emergency, and admissions occur at all times of day and night throughout the week and weekend. Following decompressive surgery the length of stay in hospital wards may be as short as one to two days, or may be longer than a week when there are ongoing bladder or bowel problems. All patients will be given adequate time to read the information leaflet with a minimum time period of six hours. Some patients will be discharged prior to being identified as being eligible for the study. These patients will be contacted by telephone by a member of the clinical team and asked if they would be willing to receive information about the study by post or email. If they agree, the information leaflet and consent form will be sent to them, and they will be re-contacted to go through the consent process over the telephone at least 24 hours after receiving the information.

All patients who are eligible for inclusion in the study will have basic anonymous clinical data collected as part of the screening log to establish participation rates and incidence at each centre. This will allow accurate assessment of the incidence of CES. Patients who do not wish to participate in the study will not be contacted further for the completion of patient reported outcome measures.

5.3 WITHDRAWAL OF STUDY PARTICIPANTS

Participants are free to withdraw from the study at any point. If withdrawal occurs, the primary reason for withdrawal will be documented in the participant's electronic case report form. The participant will not be contacted any further for outcome measures but their basic anonymous clinical details will be retained to allow accurate epidemiological assessment of the incidence of CES. If a patient loses capacity to consent for ongoing participation during the course of the study, the data they have already submitted or has already been submitted by their clinical team with their consent will continue to be used in the study, but they will not be contacted with further questionnaires.

6 DATA COLLECTION

Data relating to presentation, hospital admission, investigations, and follow up will be collected by the local trainee investigator who is a member of the clinical team caring for the patient. Data will be collected from the patient's notes, through routine interaction with the patient as part of clinical care, and through interaction with other staff members caring for the patient. All clinical and demographic information collected for this study by the local investigators is collected routinely for clinical care. No extra assessments will be performed.

Study participants who have consented to participate will also be asked to fill out details about their patient journey to the spinal unit, their symptoms, patient reported outcome measures, and service usage. These will be collected electronically anonymously via the electronic database and linked to the patient record. Patient reported outcome measures will include visual analogue scores for back and leg pain, the Oswestry Disability Index,[22] the neurogenic bowel dysfunction score,[23] the short form incontinence questionnaire,[24] the Arizona sexual experiences scale, and relevant parts of the SF-36.[25]

The timing and type of clinical reported and patient reported data that will be collected is shown in Figure 1: Study Flow Diagram.

Figure 1: Study Flow Diagram. Time points and types of data collection in UCES.

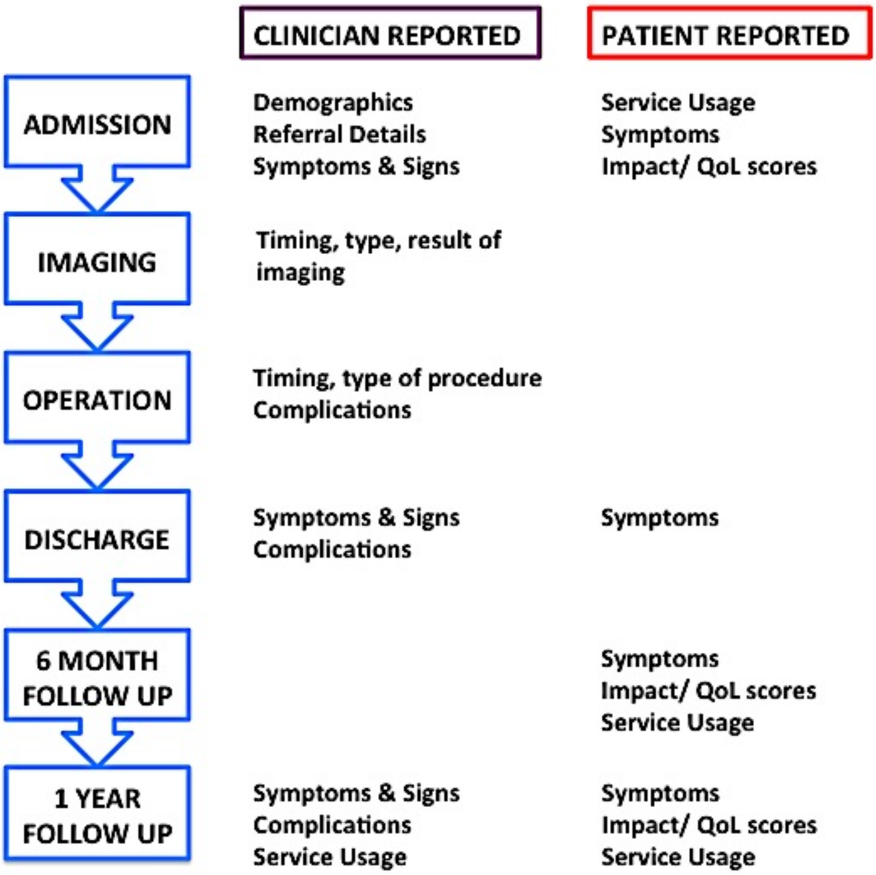

Data will be entered anonymously into a study specific secure database hosted by Castor EDC. Castor EDC complies with all applicable laws and regulations: Good Clinical Practice (GCP), European Union(EU) Annex 11, and the European Data Protection Directive. Clinician entered data will be entered directly into the database using the participant's unique study number. This study number is assigned on creating a new patient record in the database. The clinical team can only view the records of patients from their own centre. Once a participant consents for their email address to be held securely in the database, participant questionnaires are sent out via email. Each email contains a unique link for that participant with the questionnaires to complete. The responses are linked by the database to the patient's record. The participant's email address is entered by the local clinical team and this field is stored securely and is encrypted and cannot be viewed by anyone outside of the patient's local centre.

Some patients may not have an email address or may prefer to fill out questionnaires on paper. In these cases, paper questionnaires for admission and discharge time points will be provided by the local team and returned to the central study team by post identified only using the patient's unique study number.

Where patient data is routinely entered into spinal databases, surgical and outcome data from those databases will be linked anonymously to the patient record by the clinical team who routinely enter that data using the patient's unique identifier for that database or registry.

Imaging at presentation will also be reviewed on local PACS systems. We will ask for patient consent for review of imaging by the study team. All identifiable images will be stored and transferred within the NHS PACS network. Only anonymised scans will be processed outside the NHS PACS network. Anonymised imaging data will be labelled only with the study number and stored on anonymised CDs or on encrypted hard drives and processed using computers with limited access via usernames and passwords.

All local investigators will store a copy of the link between the patient's unique study number and their contact details, National Health Service (NHS) number, hospital number, Community Health Index (CHI) number, unique identifiers for spinal databases or registries, or other identifying details on a secure password protected NHS computer. Consent forms will be stored securely in a locked NHS office. No identifying information will be entered into the secure database except the email address.

When participants prefer to fill out paper questionnaires or do not respond to the email link, their contact details (name, address, telephone number) will be passed to the central study team at NHS Lothian using the NHS email system with the consent of the patient. The central study team will contact the participants to find out whether they still wish to take part in the study. Those who wish to continue with the study will be sent the questionnaires by email, by post, or they can be completed over the telephone with a member of the central study team depending on the preference of the participant. If participants do not wish to continue with the study, they will not be contacted further.

7 STATISTICS AND DATA ANALYSIS

7.1 SAMPLE SIZE CALCULATION

We plan to establish the number of patients presenting with CES in the UK over one year. We expect to identify between 600-1000 patients with CES. Our identification and recruitment rates will be checked using capture-recapture techniques using hospital discharge diagnosis coding and existing spinal registries. Previous BNTRC projects have included all UK neurosurgical units.[21]

7.2 PROPOSED ANALYSES

7.2.1 INCIDENCE OF CES

The incidence of CES will be established based on the number of patients identified at each unit and the catchment population of that unit. If all units in the UK participate, incidence will be calculated based on UK population estimates. The incidence figure will be based on all patients identified as being eligible for the study, even if they do not consent for further participation.

7.2.2 CHARACTERISTICS OF PATIENTS WITH CES

A descriptive analysis of the clinical and demographic characteristics of presenting symptoms, signs, and outcomes of patients with CES will be performed. This will be determined from both clinician reported and patient reported data. CES incidence and characteristics will be broken down into the categories of CESS, CESI, and CESR based on the clinical data. The categorical and quantitative findings on imaging will also be described.

7.2.3 METHODS OF PRESENTATION TO SPECIALIST SERVICES

Methods of patient presentation to specialist services will be described. Type, timings, and findings of investigations in patients presenting via different routes will be compared.

7.2.4 ASSESSMENT OF ADHERENCE TO QUALITY STANDARDS

The investigation and management of patients with CES will be described and compared to that laid out in the care quality standards presented in section 1.2. Proportions meeting the standards will be reported.

7.2.5 CES OUTCOMES

Patient outcomes will be assessed and analysed using both clinician and patient reported outcome measures at six months and one year. Patient outcomes will be stratified by demographics, presenting features, timing and findings of investigations, and timing and type of surgery.

7.2.6 SERVICE USAGE

Patient usage of healthcare services over the year following diagnosis and management of CES will be assessed using both patient reported service usage and electronic records.

8 OVERSIGHT ARRANGEMENTS

8.1 INSPECTION OF RECORDS

Investigators and institutions involved in the study will permit study related monitoring and audits on behalf of the sponsor, REC review, and regulatory inspection if required. In the event of audit or monitoring, the Investigator agrees to allow the representatives of the sponsor direct access to all study records and source documentation. In the event of regulatory inspection, the Investigator agrees to allow inspectors direct access to all study records and source documentation. A study specific risk assessment may be performed by representatives of the sponsor if required. The outcomes of the risk assessment will form the basis of any monitoring or audit plans.

9 GOOD CLINICAL PRACTICE

9.1 ETHICAL CONDUCT

The study will be conducted in accordance with the principles of the International Conference on Harmonisation Tripartite Guideline for Good Clinical Practice (ICH GCP). Before the study can commence, all required approvals will be obtained and any conditions of approvals will be met.

9.2 INVESTIGATOR RESPONSIBILITIES

The Investigator is responsible for the overall conduct of the study at the site and compliance with the protocol and any protocol amendments. In accordance with the principles of ICH GCP, the following areas listed in this section are also the responsibility of the Investigator. Responsibilities may be delegated to an appropriate member of study site staff.

9.2.1 INFORMED CONSENT

The Investigator is responsible for ensuring informed consent is obtained before any protocol specific procedures are carried out. The decision of a participant to participate in clinical research is voluntary and should be based on a clear understanding of what is involved. Participants must receive adequate oral and written information – appropriate Participant Information Leaflets and Informed Consent Forms will be provided. The oral explanation to the participant will be performed by the Investigator or qualified delegated person, and must cover all the elements specified in the Participant Information Leaflet and Consent Form. The participant must be given every opportunity to clarify any points they do not understand and, if necessary, ask for more information. The participant must be given sufficient time to consider the information provided. It should be emphasised that the participant may withdraw their consent to participate at any time without loss of benefits to which they otherwise would be entitled. The participant will be informed and agree to their medical records being inspected by regulatory authorities and representatives of the sponsor. The Investigator or delegated member of the study team and the participant will sign and date the informed consent form to confirm that consent has been obtained. The participant will receive a copy of this document and a copy filed in the Investigator Site File (ISF).

9.2.2 STUDY SITE STAFF

The Investigator must be familiar with the protocol and the study requirements. It is the Investigator's responsibility to ensure that all staff assisting with the study are adequately informed about the protocol and their study related duties.

9.2.3 DATA RECORDING

The Principal Investigator is responsible for the quality of the data recorded in the database at each site.

9.2.4 GCP TRAINING

All researchers are encouraged to undertake GCP training in order to understand the principles of GCP. However, this is not a mandatory requirement. GCP training status for all investigators should be indicated in their respective CVs.

9.2.5 CONFIDENTIALITY

All evaluation forms, reports, and other records must be identified in a manner designed to maintain participant confidentiality. All records must be kept in a secure storage area with limited access. Clinical information will not be released without the written permission of the participant. The Investigator and study site staff involved with this study may not disclose or use for any purpose other than performance of the study, any data, record, or other unpublished, confidential information disclosed to those individuals for the purpose of the study. Prior written agreement from the sponsor or its designee must be obtained for the disclosure of any said confidential information to other parties.

9.2.6 DATA PROTECTION

All Investigators and study site staff involved with this study must comply with the requirements of the Data Protection Act 1998 with regard to the collection, storage, processing and disclosure of personal information and will uphold the Act's core principles. Access to collated participant data will be restricted to individuals from the research team treating the participants, representatives of the sponsor and representatives of regulatory authorities. Computers used to collate the data will have limited access measures via user names and passwords. Published results will not contain any personal data that could allow identification of individual participants.

10 STUDY CONDUCT RESPONSIBILITIES

10.1 PROTOCOL AMENDMENTS

Any changes in research activity, except those necessary to remove an apparent, immediate hazard to the participant in the case of an urgent safety measure, must be reviewed and approved by the Chief Investigator. Amendments will be submitted to a sponsor representative for review and authorisation before being submitted in writing to the appropriate REC, and local R&D for approval prior to participants being enrolled into an amended protocol.

10.2 MANAGEMENT OF PROTOCOL NON COMPLIANCE

Prospective protocol deviations, i.e. protocol waivers, will not be approved by the sponsor and therefore will not be implemented, except where necessary to eliminate an immediate hazard to study participants. If this necessitates a subsequent protocol amendment, this should be submitted to the REC, and local R&D for review and approval if appropriate. Protocol deviations will be recorded in a protocol deviation log and logs will be submitted to the sponsors every 3 months. Each protocol violation will be reported to the sponsor within 3 days of becoming aware of the violation. All protocol deviation logs and violation forms should be emailed to QA@accord.scot. Deviations and violations are non-compliance events discovered after the event has occurred. Deviation logs will be maintained for each site in multi-centre studies. An alternative frequency of deviation log submission to the sponsors may be agreed in writing with the sponsors.

10.3 SERIOUS BREACH REQUIREMENTS

A serious breach is a breach which is likely to affect to a significant degree:
(a) the safety or physical or mental integrity of the participants of the trial; or

(b) the scientific value of the trial.
If a potential serious breach is identified by the Chief investigator, Principal Investigator or delegates, the sponsors (seriousbreach@accord.scot) must be notified within 24 hours. It is the responsibility of the co-sponsors to assess the impact of the breach on the scientific value of the trial, to determine whether the incident constitutes a serious breach and report to research ethics committees as necessary.

10.4 STUDY RECORD RETENTION

All study documentation will be kept for a minimum of 5 years from the protocol defined end of study point. When the minimum retention period has elapsed, study documentation will not be destroyed without permission from the sponsor.

10.5 END OF STUDY

The end of the study is 18 months after the enrolment of the last participant. The Investigators or the sponsor have the right at any time to terminate the study for clinical or administrative reasons. The end of the study will be reported to the REC, and R&D Offices and sponsor within 90 days, or 15 days if the study is terminated prematurely. The Investigators will inform participants of the premature study closure. End of study notification will be reported to the sponsor via email to resgov@accord.scot. A summary report of the study will be provided to the REC within 1 year of the end of the study.

10.6 INSURANCE AND INDEMNITY

The sponsor is responsible for ensuring proper provision has been made for insurance or indemnity to cover their liability and the liability of the Chief Investigator and staff. The following arrangements are in place to fulfil the sponsor's responsibilities:

- Sites participating in the study will be liable for clinical negligence and other negligent harm to individuals taking part in the study and covered by the duty of care owed to them by the sites concerned. The sponsor requires individual sites participating in the study to arrange for their own insurance or indemnity in respect of these liabilities. Sites which are part of the United Kingdom's National Health Service will have the benefit of NHS Indemnity.

11 REPORTING, PUBLICATIONS AND NOTIFICATION OF RESULTS

11.1 AUTHORSHIP POLICY

Ownership of the complete dataset arising from this study resides with the steering committee and the BNTRC. On completion of the study, the data will be analysed and tabulated, and a report will be prepared. Local data collected as part of this study belongs to the local team collecting that data.

11.2 PUBLICATION

The study report will be used for publication and presentation at scientific meetings. Investigators have the right to publish orally or in writing the results of the study. Summaries of results will also be made available to investigators. Following the initial analysis and publication, study data will be made available to those who submit successful peer-reviewed proposals for use of the data to the steering committee via the BNTRC.

All local investigators who enter data for at least one case will be named as contributors on all publications arising from this study and will receive a certificate of collaboration in this study. Authorship of publications arising from this study will be determined in accordance with the guidelines of the International Committee of Medical Journal Editors (ICMJE).[26]

11.3 PEER REVIEW

The concept for this study was selected by a panel of judges in an open competition for support from the BNTRC. The protocol has been approved by the steering committee listed at the beginning of this protocol prior to the start of data collection.

12 REFERENCES

1. Todd NV, Dickson RA. Standards of care in cauda equina syndrome. *British Journal of Neurosurgery.* 2016;30(5):518-522.
2. Fraser S, Roberts L, Murphy E. Cauda equina syndrome: a literature review of its definition and clinical presentation. *Archives of Physical Medicine and Rehabilitation.* 2009;90(11):1964-1968.
3. Balasubramanian K, Kalsi P, Greenough CG, Kuskoor Seetharam MP. Reliability of clinical assessment in diagnosing cauda equina syndrome. *British Journal of Neurosurgery.* 2010;24(4):383-386.
4. Todd NV. Causes and outcomes of cauda equina syndrome in medico-legal practice: a single neurosurgical experience of 40 consecutive cases. *British Journal of Neurosurgery.* 2011;25(4):503-508.
5. Quraishi NA, Hammett TC, Todd DB, Bhutta MA, Kapoor V. Malpractice litigation and the spine: the NHS perspective on 235 successful claims in England. *European Spine Journal.* 2012;21 Suppl 2:S196-199.
6. Germon T, Ahuja S, Casey AT, Todd NV, Rai A. British Association of Spine Surgeons standards of care for cauda equina syndrome. *The Spine Journal.* 2015;15(3 Suppl):S2-4.
7. Society of British Neurological Surgeons. Standards of Care for Established and Suspected Cauda Equina Syndrome. 2009, version 4.
8. Society of British Neurological Surgeons. SBNS Care Quality Statement. 2015.
9. Ahn UM, Ahn NU, Buchowski JM, Garrett ES, Sieber AN, Kostuik JP. Cauda equina syndrome secondary to lumbar disc herniation: a meta-analysis of surgical outcomes. *Spine.* 2000;25(12):1515-1522.
10. Kohles SS, Kohles DA, Karp AP, Erlich VM, Polissar NL. Time-dependent surgical outcomes following cauda equina syndrome diagnosis: comments on a meta-analysis. *Spine.* 2004;29(11):1281-1287.
11. Korse NS, Jacobs WC, Elzevier HW, Vleggeert-Lankamp CL. Complaints of micturition, defecation and sexual function in cauda equina syndrome due to lumbar disk herniation: a systematic review. *European Spine Journal.* 2013;22(5):1019-1029.
12. Gardner A, Gardner E, Morley T. Cauda equina syndrome: a review of the current clinical and medico-legal position. *European Spine Journal.* 2011;20(5):690-697.
13. Bell DA, Collie D, Statham PF. Cauda equina syndrome: what is the correlation between clinical assessment and MRI scanning? *British Journal of Neurosurgery.* 2007;21(2):201-203.
14. Gleave JR, Macfarlane R. Cauda equina syndrome: what is the relationship between timing of surgery and outcome? *British Journal of Neurosurgery.* 2002;16(4):325-328.
15. Srikandarajah N, Boissaud-Cooke MA, Clark S, Wilby MJ. Does early surgical decompression in cauda equina syndrome improve bladder outcome? *Spine.* 2015;40(8):580-583.
16. Crocker M, Fraser G, Boyd E, Wilson J, Chitnavis BP, Thomas NW. The value of interhospital transfer and emergency MRI for suspected cauda equina syndrome: a 2-year retrospective study. *Annals of the Royal College of Surgeons of England.* 2008;90(6):513-516.
17. Cauda equina syndrome. *Medical Protection Society UK Casebook.* 2000;20:9-13.
18. Schoenfeld AJ. Incidence and Epidemiology of Cauda Equina Syndrome: A Review of 976 Patients from a Complete American Population. *Proceedings of the NASS 27th Annual Meeting.* Vol 12: The Spine Journal; 2012:99S-165S.
19. The National Spinal Taskforce. *Commissioning Spinal Services - Getting The Service Back On Track.* January 2013.
20. Office for National Statistics. *Annual Mid-year Population Estimates, 2010.* June 2011
21. Brennan PM, Kolias AG, Joannides AJ, et al. The management and outcome for patients with chronic subdural hematoma: a prospective, multicenter, observational cohort study in the United Kingdom. *Journal of Neurosurgery.* 2016:1-8.
22. Fairbank JC, Pynsent PB. The Oswestry Disability Index. *Spine.* 2000;25(22):2940-2952
23. Krogh K, Christensen P, Sabroe S, Laurberg S. Neurogenic bowel dysfunction score. *Spinal Cord.* 2006;44(10):625-631.
24. McGrother CW, Donaldson MM, Shaw C, et al. Storage symptoms of the bladder: prevalence, incidence and need for services in the UK. *BJU International.* 2004;93(6):763-769.
25. McGahuey CA, Gelenberg AJ, Laukes CA, et al. The Arizona Sexual Experience Scale (ASEX): reliability and validity. *Journal of Sex & Marital Therapy.* 2000;26(1):25-40.
26. International Committee of Medical Journal Editors. Recommendations for the Conduct, Reporting, Editing and Publication of Scholarly Work in Medical Journals.
